# LsAP2 regulates leaf morphology by inhibiting CIN-like TCP transcription factors and repressing *LsKAN2* in lettuce

**DOI:** 10.1038/s41438-021-00622-y

**Published:** 2021-09-01

**Authors:** Chen Luo, Shenglin Wang, Kang Ning, Zijing Chen, Yixin Wang, Jingjing Yang, Qian Wang

**Affiliations:** Beijing Key Laboratory of Growth and Developmental Regulation for Protected Vegetable Crops, Department of Vegetable Science, College of Horticulture, China Agricultural University, Beijing, 100193 China

**Keywords:** Leaf development, Plant molecular biology, Plant polarity, Reporter genes, Reverse transcription polymerase chain reaction

## Abstract

Leaf size and flatness directly affect photosynthesis and are closely related to agricultural yield. The final leaf size and shape are coordinately determined by cell proliferation, differentiation, and expansion during leaf development. Lettuce (*Lactuca sativa* L.) is one of the most important leafy vegetables worldwide, and lettuce leaves vary in shape and size. However, the molecular mechanisms of leaf development in lettuce are largely unknown. In this study, we showed that the lettuce *APETALA2* (*LsAP2*) gene regulates leaf morphology. *LsAP2* encodes a transcriptional repressor that contains the conserved EAR motif, which mediates interactions with the TOPLESS/TOPLESS-RELATED (TPL/TPR) corepressors. Overexpression of *LsAP2* led to small and crinkly leaves, and many bulges were seen on the surface of the leaf blade. LsAP2 physically interacted with the CINCINNATA (CIN)-like TEOSINTE BRANCHED1/CYCLOIDEA/PROLIFERATING CELL FACTOR (TCP) transcription factors and inhibited their transcriptional activation activity. RNA sequencing analysis showed that LsAP2 affected the expression of auxin- and polarity-related genes. In addition, LsAP2 directly repressed the abaxial identity gene *KANADI2* (*LsKAN2*). Together, these results indicate that LsAP2 regulates leaf morphology by inhibiting CIN-like TCP transcription factors and repressing *LsKAN2*, and our work provides insights into the regulatory mechanisms of leaf development in lettuce.

## Introduction

Leaves are important plant organs in which photosynthesis converts carbon dioxide and water to carbohydrates and oxygen^1^. Leaf size is crucial for photosynthesis because leaf area directly affects light absorption^2^. Most plants have evolved flat leaves to efficiently capture light energy^3^. Large and flat leaves photosynthesize strongly and synthesize more organic compounds than other leaf types^4^. Because leaf size and flatness are closely related to agricultural yield, understanding the regulatory mechanisms and genetic bases of leaf development will contribute to the improvement of crop production.

Leaf development is initiated from the shoot apical meristem and is controlled by complex regulatory mechanisms^5^. Initially, leaf primordia are specified in the flanking region of the shoot apical meristem. Subsequently, the adaxial–abaxial and proximal–distal axes are established with the bulging of the leaf primordia. Then, the blade and petiole regions are specified. After leaf blade formation, cell proliferation and differentiation occur throughout the leaf blade. Finally, the leaves become fully developed, revealing their specific sizes and shapes^2,3^.

Establishment of leaf adaxial–abaxial polarity is required for the flat outgrowth of the lamina^6^. In *Arabidopsis*, the processes involved in establishing adaxial–abaxial polarity have been studied extensively. The classic two-domain view is that a leaf primordium can be divided into two domains, namely, an adaxial domain and an abaxial domain, that can suppress each other. *PHABULOSA* (*PHB*), *PHAVOLUTA* (*PHV*), and *REVOLUTA* (*REV*), which encode the class III HD-ZIP transcription factors, define the adaxial domain^7^, which is also promoted by two genes, namely, *ASYMMETRIC LEAVES1* (*AS1*) and *ASYMMETRIC LEAVES2* (*AS2*)^8^. Members of the KANADI (KAN) transcription factor family, namely, *KAN1* and *KAN2*, together with *AUXIN RESPONSE FACTOR3* (*ARF3*) and *ARF4*, promote the abaxial domain^9,10^. In addition, members of the YABBY (YAB) transcription factor family, such as *YAB1*, *YAB2*, and *YAB3*, redundantly promote the abaxial domain^11^.

Leaf size and shape are determined by strict control of cell proliferation, differentiation, and expansion during leaf development^2,3^. The class II TEOSINTE BRANCHED1/CYCLOIDEA/PROLIFERATING CELL FACTOR (TCP) transcription factors are well-known regulators of leaf development and play dominant roles in modulating leaf size and morphology^12^. The TCP transcription factors repress the activity of the marginal meristem and promote the switch from cell proliferation to cell differentiation^13,14^. Inactivation of *CINCINNATA* (*CIN*), which encodes a TCP protein in snapdragon (*Antirrhinum majus* L.), leads to delayed cell differentiation, and the leaves of *cin* mutants are crinkly and display excessive growth in the interveinal and marginal regions^15^. The functions of CIN-like TCP transcription factors (CIN-like TCPs) during leaf development appear to be conserved across diverse plant species, because disruption of CIN-like TCPs in *Arabidopsis* and tomato (*Solanum lycopersicum* L.) also caused abnormal morphology^16–18^.

Lettuce (*Lactuca sativa* L.) belongs to the large Asteraceae family and is one of the most popular leafy vegetables worldwide^19^. Lettuce cultivars can be classified into several horticultural types according to their morphological characteristics, and most of the cultivars are leafy types that are harvested for their leaves^20^. Leafy lettuce supplies energy, vitamins, dietary fibers, and minerals and is mostly consumed as a fresh vegetable^21^. Recently, transgenic lettuce expressing small artificial RNA was found to be useful in the treatment of hepatitis B virus infection^22^, indicating that lettuce could be a natural manufacturer of both food and medicine and opening up new possibilities for the lettuce industry in the future. Lettuce leaves vary in shape, size, and color^23–25^, but the molecular mechanisms of leaf development in lettuce are largely unknown.

In a previous study, we investigated the role of lettuce APETALA2 (LsAP2) in regulating seed shape^26^. In this study, we show that LsAP2 also functions as a regulator of leaf development and describe LsAP2-related regulatory networks that may be involved in leaf development. Our results will not only advance the understanding of the mechanism of LsAP2 in regulating leaf morphology but also provide insights into the molecular regulatory networks of leaf development in lettuce.

## Results

### *LsAP2* is expressed in leaves and shoot apexes

**Fig. 1:**
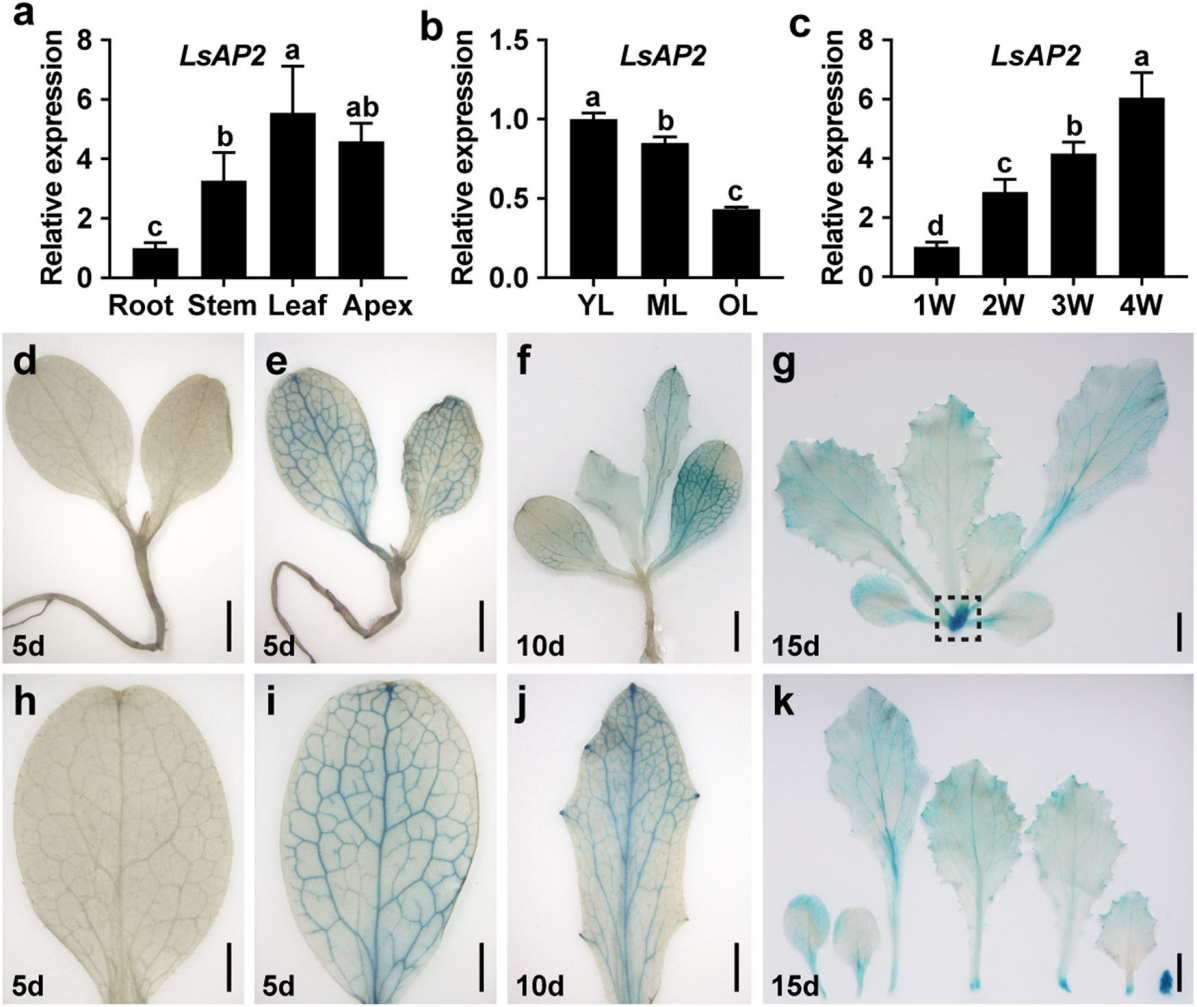
*LsAP2* is expressed in leaves and shoot apexes. **a** Expression of *LsAP2* in different vegetative tissues. The values are means ± SDs (*n* = 3). The data were normalized to a value of 1 for the root. **b** Expression of *LsAP2* in different leaves. YL young leaf, ML mature leaf, OL old leaf. The values are means ± SDs (*n* = 3). The data were normalized to a value of 1 for the young leaf. **c** Expression of *LsAP2* in shoot apexes at different vegetative growth stages. 1 W, 1 week; 2 W, 2 weeks; 3 W, 3 weeks; 4 W, 4 weeks. The values are means ± SDs (*n* = 3). The data were normalized to a value of 1 for the 1-week-old shoot apex. **d** A 5-d-old wild type (WT) seedling. **e** A 5-d-old *pLsAP2:GUS* seedling. **f** A 10-d-old *pLsAP2:GUS* seedling. **g** A 15-d-old *pLsAP2:GUS* seedling. The shoot apex is shown in the dotted box. **h** Cotyledon of a 5-d-old WT seedling. **i** Cotyledon of a 5-d-old *pLsAP2:GUS* seedling. **j** True leaf of a 10-d-old *pLsAP2:GUS* seedling. **k** Leaves and shoot apex of a 15-d-old *pLsAP2:GUS* seedling. In (**a**–**c**), different letters indicate significant differences determined by one-way ANOVA with Duncan’s post hoc test (*P* < 0.05). Scale bars: (**d**–**f**) 2 mm; (**g**, **k**) 5 mm; (**h**–**j**) 1 mm

*APETALA2* (*AP2*) encodes a member of the large APETALA2/ETHYLENE RESPONSIVE FACTOR (AP2/ERF) transcription factor family, and the AP2 protein is involved in various developmental processes in many plant species^27^. To determine the potential functions of *LsAP2* during vegetative growth in lettuce, we explored its expression patterns in vegetative tissues by quantitative real-time PCR (qRT-PCR). The expression of *LsAP2* was higher in the leaf and shoot apex than in the root and stem (Fig. 1a). In addition, *LsAP2* exhibited higher expression in young and mature leaves than in old leaves (Fig. 1b). Because the shoot apex supports the vertical growth of lettuce and gives rise to all other lateral meristems and organs^28^, we also investigated the expression of *LsAP2* in shoot apexes at different vegetative growth stages. We found that *LsAP2* expression gradually increased between successive stages of vegetative growth (Fig. 1c).

To examine the promoter activity of *LsAP2*, we performed GUS (β-glucuronidase) staining analysis on *pLsAP2:GUS* plants. We detected GUS activity in cotyledons, leaves, and shoot apexes (Fig. 1e–g). Interestingly, the promoter activity of *LsAP2* was detected in leaf veins, because strong GUS signals were found in the venation of the cotyledon (Fig. 1i) and in the developing leaves (Fig. 1j, k). As the plants developed, GUS staining gradually faded from the leaf medial to marginal regions, and strong GUS activity was observed in the leaf axils and shoot apex (Fig. 1k). Together, these results showed that *LsAP2* was expressed in leaves and shoot apexes, suggesting that LsAP2 may play roles during the vegetative growth of lettuce.

### *LsAP2* encodes an EAR motif-containing transcriptional repressor

**Fig. 2:**
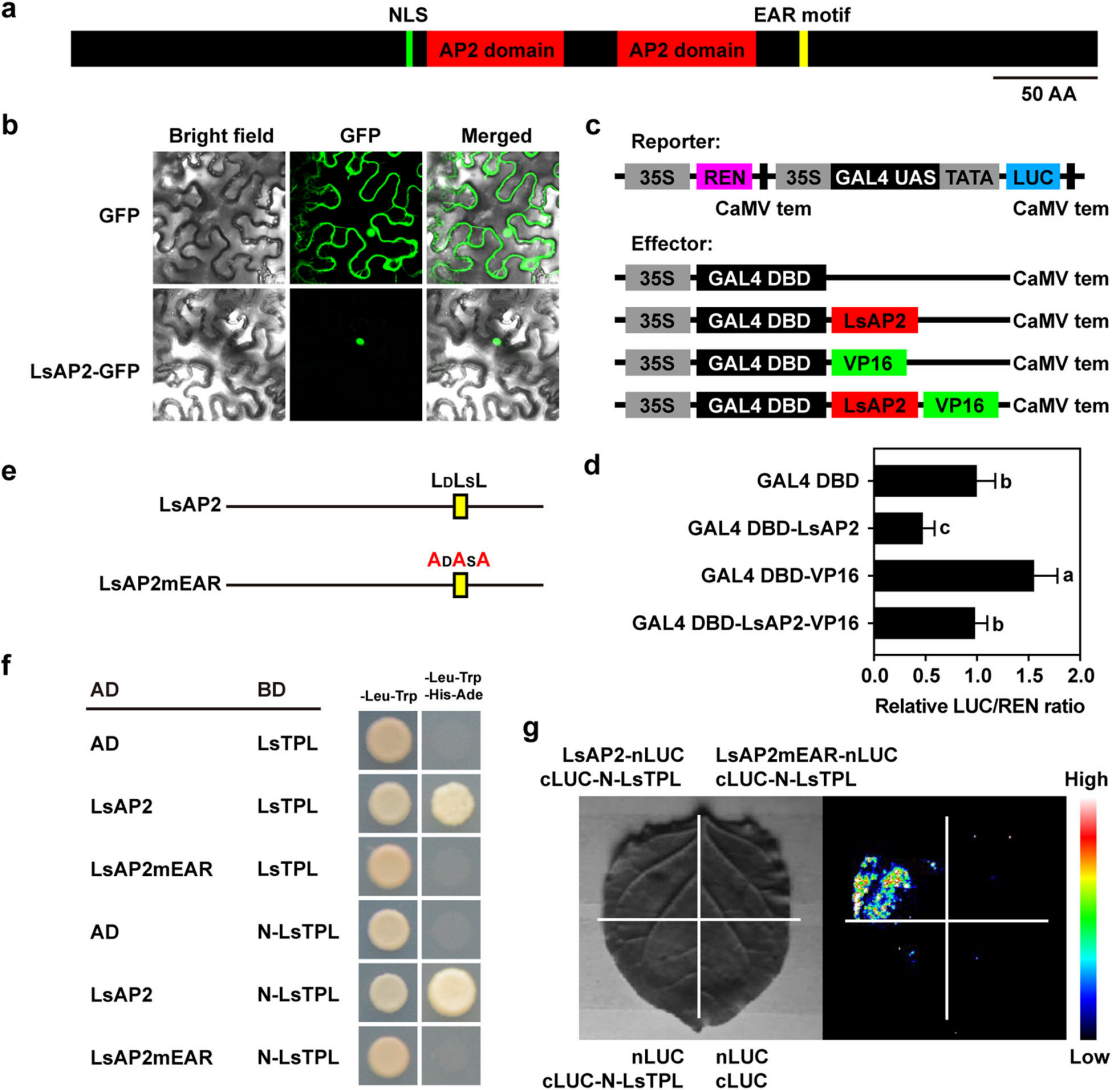
*LsAP2* encodes an EAR motif-containing transcriptional repressor. **a** Schematic representation of the LsAP2 protein structure. NLS nuclear localization sequence, EAR ethylene-responsive element binding factor-associated amphiphilic repression, AA amino acid. **b** Subcellular localization of LsAP2. **c** Schematic representation of the reporter and effector constructs used in the transcriptional activity assays. **d** Measurement of the relative firefly luciferase/Renilla luciferase (LUC/REN) ratio after transient coexpression of the reporter and effector constructs into tobacco leaves. The values are means ± SDs (*n* = 6). The data were normalized to a value of 1 for the GAL4 DBD group. Different letters indicate significant differences determined by one-way ANOVA with Tukey’s post hoc test (*P* < 0.05). **e** Mutation of the EAR motif (mEAR) in the LsAP2 protein sequence. **f** Yeast two-hybrid assays showing that LsAP2 interacts with LsTPL through the EAR motif. AD activation domain, BD binding domain. **g** Luciferase complementation imaging (LCI) assays showing the interaction between LsAP2 and N-LsTPL in tobacco leaves

The deduced LsAP2 protein sequence contained a potential nuclear localization sequence (NLS) in the N-terminal region (Fig. 2a; Fig. S1). To determine the subcellular localization of LsAP2, the LsAP2 protein was fused with green fluorescent protein (GFP) under the control of the Super promoter. Transient expression of the *Super:LsAP2-GFP* construct into tobacco (*Nicotiana benthamiana*) leaves showed that LsAP2 was localized to the nucleus (Fig. 2b), indicating that LsAP2 was a nuclear protein.

We also found that a typical ethylene-responsive element binding factor-associated amphiphilic repression (EAR) motif was located at the C-terminus of the LsAP2 protein (Fig. 2a; Fig. S1). Because EAR motif-containing proteins play essential roles in diverse biological processes by negatively regulating gene expression^29^, we speculated that LsAP2 might function as a transcriptional repressor. To test this idea, we performed transcriptional activity assays using the GAL4/UAS system (Fig. 2c). We found that leaves expressing GAL4 DBD-LsAP2 had a lower relative firefly luciferase/Renilla luciferase (LUC/REN) ratio and leaves expressing GAL4 DBD-VP16 had a higher relative LUC/REN ratio than the control group, which expressed GAL4 DNA-binding domain (GAL4 DBD) (Fig. 2d), indicating that LsAP2 had transcriptional repression activity. In addition, when leaves expressed GAL4 DBD-LsAP2-VP16, the measured relative LUC/REN ratio was lower than that in leaves expressing GAL4 DBD-VP16 (Fig. 2d), indicating that LsAP2 inhibited the transcriptional activation activity of VP16. These results confirmed that LsAP2 was a typical transcriptional repressor.

The EAR motif has been implicated in the physical recruitment of the TOPLESS (TPL) corepressor by various EAR motif-containing proteins^30^. We identified the lettuce TPL (LsTPL) and TPL-RELATED (LsTPR) proteins by sequence and phylogenetic analyses (Fig. S2a, b). Furthermore, we showed that LsAP2 physically interacted with LsTPL by yeast two-hybrid assays (Fig. 2f) and that mutation of the EAR motif in LsAP2 abolished the interaction between LsAP2 and LsTPL (Fig. 2e, f), indicating that the EAR motif was necessary for the LsAP2–LsTPL interaction. The interactions between TPL and EAR motif-containing proteins have been shown to depend on the N-terminal region of TPL^31,32^. In this study, we confirmed that LsAP2 interacted with the N-terminal region of LsTPL (N-LsTPL) (Fig. 2f). We then investigated the interaction between LsAP2 and LsTPL in planta by luciferase complementation imaging (LCI) assays. Fluorescence was observed in the leaf area after transient coexpression of the LsAP2-nLUC and cLUC-N-LsTPL constructs in tobacco leaves (Fig. 2g), further confirming the interaction between LsAP2 and LsTPL. We also demonstrated that LsAP2 interacted with LsTPR proteins, including LsTPR1, LsTPR3, LsTPR4a, and LsTPR4b (Fig. S2c, d). These results suggest that LsAP2 may recruit TPL/TPR corepressors to form transcriptional repressor complexes in lettuce.

### Overexpression of *LsAP2* leads to small and crinkly leaves

**Fig. 3:**
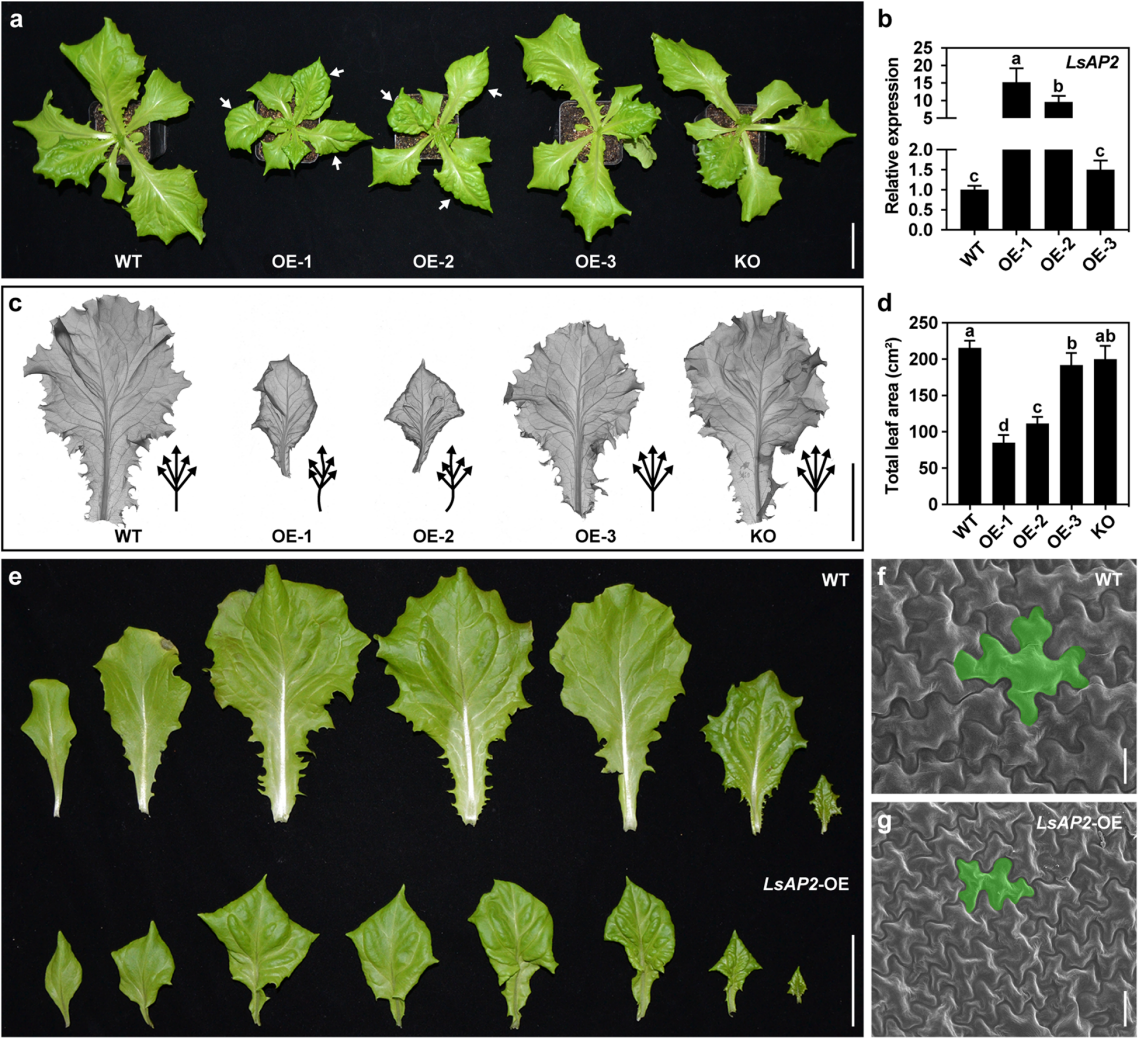
Overexpression of *LsAP2* leads to small and crinkly leaves. **a** Plant morphology of the WT, three *LsAP2* overexpression (*LsAP2*-OE) lines, and an *LsAP2* knockout (*LsAP2*-KO) line. White arrows indicate the leaves curled downwards with bulges. **b** Expression of *LsAP2* in different lines. The values are means ± SDs (*n* = 3). The data were normalized to a value of 1 for the WT. **c** Leaf venation patterns in different lines. Black arrows, schematic representation of the primary leaf veins. **d** Total leaf area of one-month-old plants in different lines. The values are means ± SDs (*n* = 10). **e** Adaxial surface of leaves from the WT and *LsAP2*-OE plants. **f**, **g** Scanning electron micrographs of adaxial epidermal cells of mature leaves from WT (**f**) and *LsAP2*-OE (**g**) plants. In (**b**) and (**d**), different letters indicate significant differences determined by one-way ANOVA with Tukey’s post hoc test (*P* < 0.05). Scale bars: (**a**, **c**, **e**) 5 cm; (**f**, **g**) 20 μm

In a previous study, we showed that *LsAP2* was expressed in floral organs and seeds and that knockout of *LsAP2* led to longer and narrower seeds in lettuce^26^. However, except for a change in seed shape, no obvious phenotype was observed in the *LsAP2* knockout plants. To gain further insight into the function of *LsAP2* in lettuce, we generated an *LsAP2* overexpression (*LsAP2*-OE) construct and obtained 13 *LsAP2*-OE lines by *Agrobacterium*-mediated transformation of lettuce. Three representative lines with different phenotype severities were chosen for further characterization (Fig. 3a). qRT-PCR analysis showed that there were more *LsAP2* transcripts in the *LsAP2*-OE plants than in the wild type (WT) and that *LsAP2* expression increased by more than 10-fold in the severely affected *LsAP2*-OE plants (Fig. 3b).

Unlike the mature leaves of the WT plants, which were large and flat, the mature leaves of *LsAP2*-OE plants were small and crinkly (Fig. 3a, c). Although a frameshift mutation and premature stop codon in the *LsAP2* knockout allele led to the absence of a functional LsAP2 protein (Fig. S3), the leaves of the *LsAP2* knockout plants looked normal, with no significant difference in leaf size or shape between WT and *LsAP2* knockout plants (Fig. 3a, c). In the *LsAP2*-OE plants, the leaf venation patterns were distorted, and the leaves became asymmetrical (Fig. 3c). Furthermore, the total leaf area of one-month-old *LsAP2*-OE plants was significantly less than that of one-month-old WT plants (Fig. 3d).

In severely affected *LsAP2*-OE plants, the leaves curled downwards and displayed excessive growth in the interveinal regions, and many bulges were seen on the surface of the leaf blade (Fig. 3e). These changes in leaf morphology appeared in the third true leaf and became more pronounced in the subsequent leaves. The interveinal overgrowth and distorted leaf venation patterns of *LsAP2*-OE plants corresponded to *LsAP2* expression in leaf veins. Scanning electron microscopy images of the epidermal cells from mature leaves showed that the leaf epidermal cells of *LsAP2*-OE plants were much smaller than those of the WT (Fig. 3f, g), suggesting that LsAP2 may affect cell expansion during leaf development. Together, these data indicated that LsAP2 functions as a negative regulator of leaf development.

### LsAP2 interacts with CIN-like TCPs

**Fig. 4:**
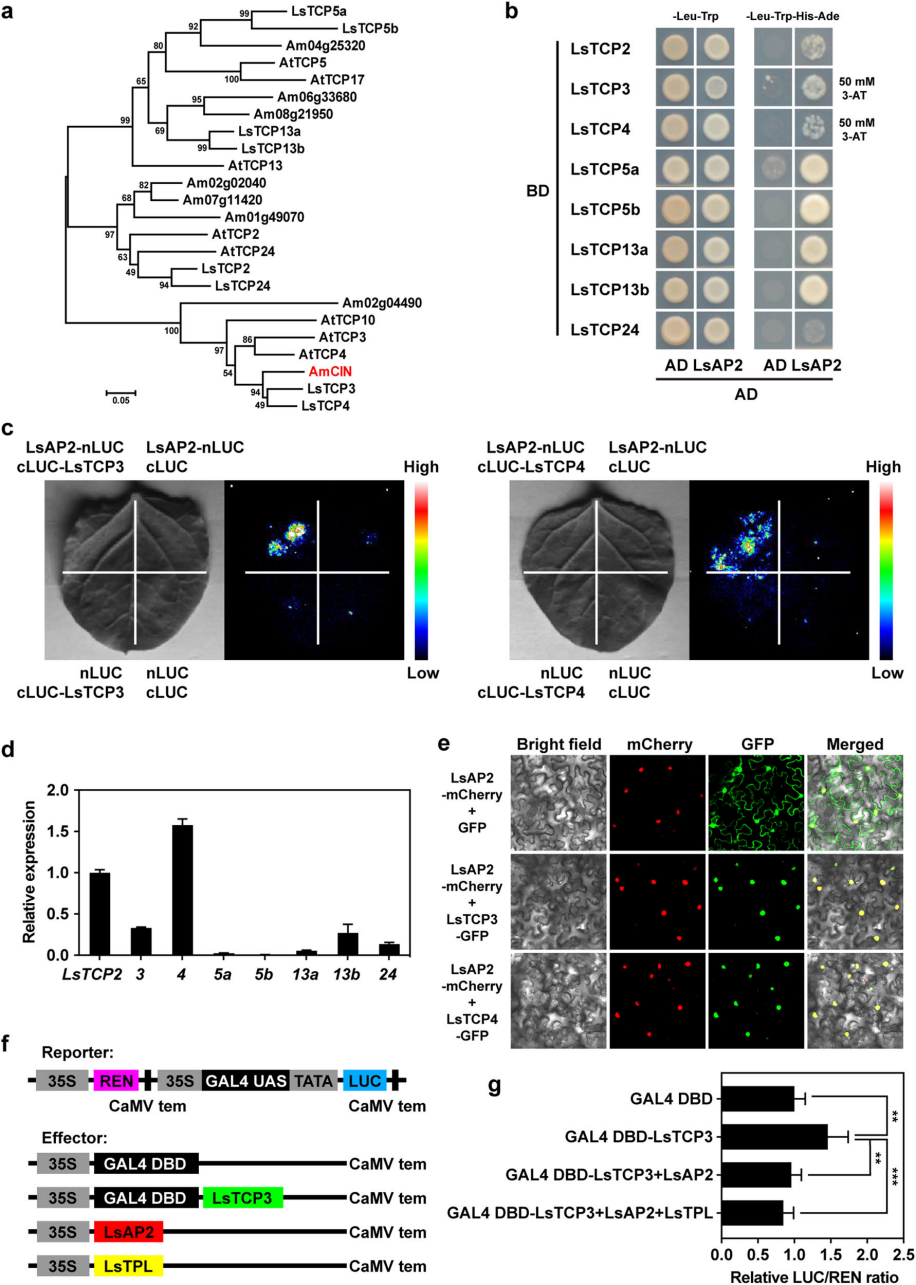
LsAP2 interacts with CIN-like TCPs and inhibits their transcriptional activation activity. **a** Phylogenetic analysis of CIN-like TCP proteins. Ls *Lactuca sativa*, Am *Antirrhinum majus*, At *Arabidopsis thaliana*. The *Antirrhinum* CIN is indicated in red font. **b** Yeast two-hybrid assays showing the interactions between LsAP2 and CIN-like TCPs. We used 50 mM 3-amino-1,2,4-triazole (3-AT) for LsTCP3 and LsTCP4 and 0 mM 3-AT for the other LsTCPs. **c** LCI assays showing that LsAP2 interacts with LsTCP3 and LsTCP4. **d** Expression of *CIN-like TCP* genes in leaves. The values are means ± SDs (*n* = 3). The expression data of *LsTCP2* in leaves were normalized to 1. **e** Subcellular localization of LsTCP3 and LsTCP4 proteins. **f** Schematic representation of the reporter and effector constructs used in the transcriptional activity assays. **g** Measurement of the relative LUC/REN ratio after transient coexpression of the reporter and effector constructs in tobacco leaves. The values are means ± SDs (*n* = 6). The data were normalized to a value of 1 for the GAL4 DBD group. Significant differences were determined by Student’s *t* test (**P < 0.01; ***P < 0.001)

The leaf morphology of *LsAP2*-OE plants was similar to that of the loss-of-function mutants of *CIN* in *Antirrhinum*, in which the leaves were also crinkly and had bulges between veins^15^. The functions of CIN-like TCPs during leaf development appear to be conserved across diverse plant species^33^. We speculated that LsAP2 might be associated with CIN-like TCPs. To test this idea, we identified eight lettuce CIN-like TCPs by sequence and phylogenetic analyses (Fig. 4a; Fig. S4), and our results showed that LsTCP3 and LsTCP4 were closely related to *Antirrhinum* CIN.

Previous studies revealed that EAR motif-containing repressors can physically interact with CIN-like TCPs and control their activity^32,34^. We considered that LsAP2 might directly interact with CIN-like TCPs and affect their functions. To investigate this further, we performed yeast two-hybrid assays and confirmed that LsAP2 interacted with CIN-like TCPs (Fig. 4b). Our results showed that the interactions between LsAP2 and LsTCP3, LsTCP4, LsTCP5a, LsTCP5b, LsTCP13a, or LsTCP13b were strong, but those between LsAP2 and LsTCP2 or LsTCP24 were weak (Fig. 4b). We also investigated the interactions between LsAP2 and CIN-like TCPs in planta by LCI assays. The results confirmed that LsAP2 physically interacted with CIN-like TCPs (Fig. 4c; Fig. S5), which was consistent with the results of the yeast two-hybrid assays.

### LsAP2 inhibits the transcriptional activation activity of CIN-like TCPs

To test whether lettuce CIN-like TCPs are involved in leaf development, we analyzed the expression of *CIN-like TCP* genes during leaf development. We found that most of the *CIN-like TCP* genes were expressed in leaves, and the expression levels of *LsTCP2*, *LsTCP3*, and *LsTCP4* were higher than those of the other *CIN-like TCP* genes (Fig. 4d). Because the nuclear localization of some CIN-like TCPs has been reported^35,36^, we performed transient expression assays to determine the subcellular localization of lettuce CIN-like TCPs. The CIN-like TCPs were fused with GFP under the control of the Super promoter, and the LsAP2 protein was fused with the red fluorescent protein mCherry under the control of the Super promoter as a nuclear marker. Our results demonstrated that lettuce CIN-like TCPs were localized to the nucleus and overlapped with LsAP2 (Fig. 4e; Fig. S6), which indicated that the lettuce CIN-like TCPs were nuclear proteins.

We speculated that LsAP2 might function as a repressor to inhibit the transcriptional activation activity of CIN-like TCPs. To test this idea, we performed transcriptional activity assays for LsTCP3 (Fig. 4f). Compared with the control group that expressed GAL4 DBD, the leaves expressing GAL4 DBD-LsTCP3 had a higher relative LUC/REN ratio (Fig. 4g), indicating that LsTCP3 was a transcriptional activator. Leaves expressing GAL4 DBD-LsTCP3 and LsAP2 together had a lower relative LUC/REN ratio than those expressing GAL4 DBD-LsTCP3 (Fig. 4g), suggesting that LsAP2 inhibited the transcriptional activation activity of LsTCP3. When we also transformed the LsTPL corepressor, the measured relative LUC/REN ratio decreased further (Fig. 4g). Together, these results demonstrated that LsAP2 interacted with CIN-like TCPs and inhibited their transcriptional activation activity.

### LsAP2 affects the expression of auxin- and polarity-related genes

**Fig. 5:**
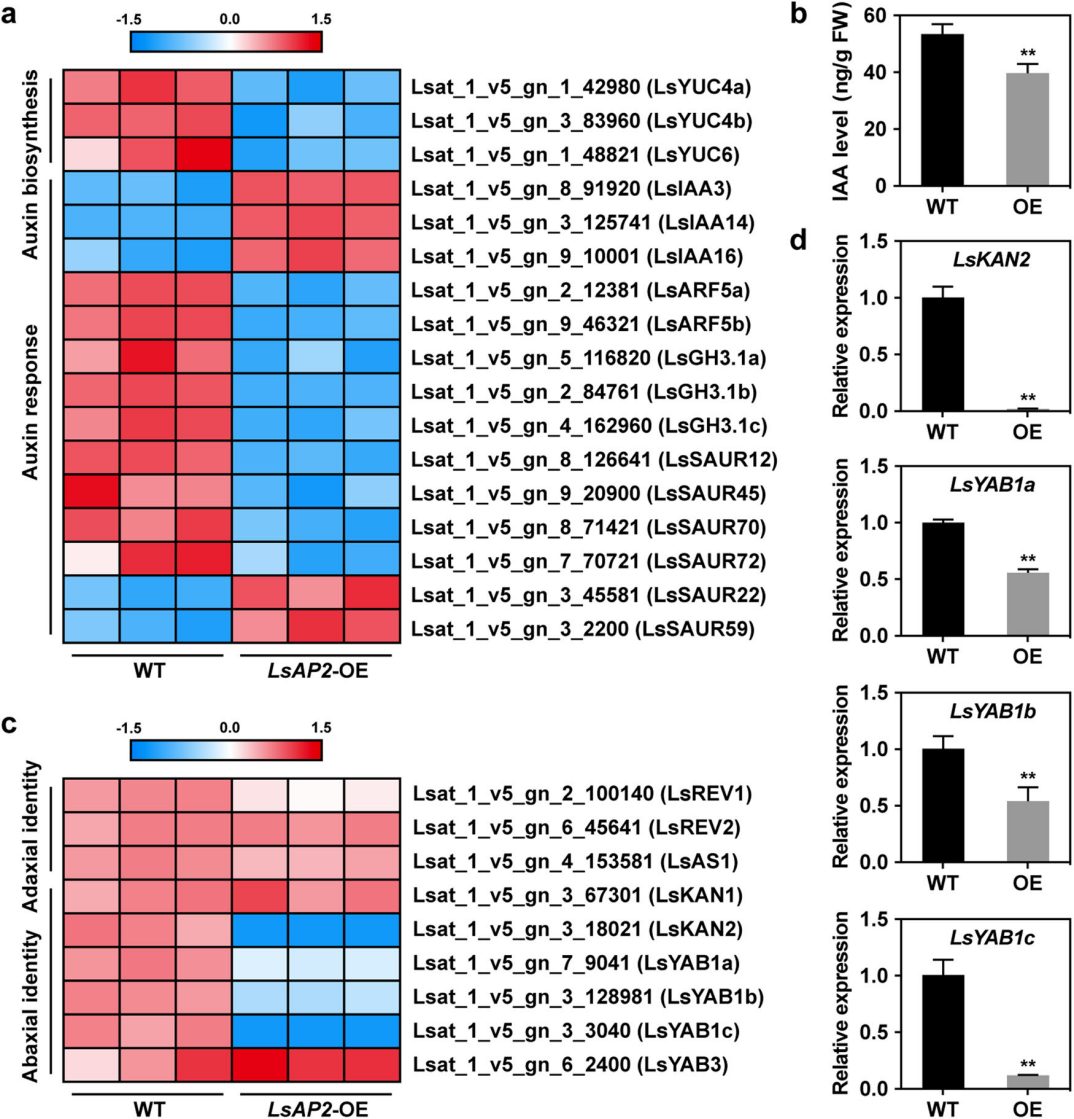
LsAP2 affects the expression of auxin- and polarity-related genes. **a** Heat map showing the expression levels of auxin-related genes between WT and *LsAP2*-OE plants. Red and blue indicate up- and downregulation, respectively. **b** Indole-3-acetic acid (IAA) level in the leaves of WT and *LsAP2*-OE plants. The values are means ± SDs (*n* = 3). **c** Heat map showing the expression levels of adaxial–abaxial identity-related genes between WT and *LsAP2*-OE plants. **d** qRT-PCR analysis showing the expression levels of abaxial identity-related genes between WT and *LsAP2*-OE plants. The values are means ± SDs (*n* = 3). The data were normalized to a value of 1 for the WT. In (**b**) and (**d**), significant differences were determined by Student’s *t* test (**P < 0.01)

To further explore how LsAP2 regulates leaf morphology, we performed RNA sequencing (RNA-seq) analysis of the leaves from WT and *LsAP2*-OE plants. A total of 2579 differentially expressed genes were identified between WT and *LsAP2*-OE plants (Dataset S1). Kyoto Encyclopedia of Genes and Genomes (KEGG) analysis of the differentially expressed genes revealed that genes related to plant hormone signal transduction were significantly enriched (Fig. S7a), and most of them were auxin response genes (Fig. 5a). *AUXIN*/*INDOLE-3-ACETIC ACID* (*AUX*/*IAA*) genes, which are negative regulators of auxin signaling^37^, were upregulated, whereas *ARF5* and *GH3* genes were downregulated, in *LsAP2*-OE plants compared with WT. ARF5 is a major activator that mediates the auxin transcriptional response^38^, and GH3 proteins play roles in auxin homeostasis^39^. Many *SMALL AUXIN UP RNA* (*SAUR*) genes, which are the largest family of early auxin response genes^40^, were also differentially expressed between WT and *LsAP2*-OE plants. In addition, the expression levels of three *YUCCA* (*YUC*) auxin biosynthesis genes were decreased in *LsAP2*-OE plants (Fig. 5a), and consistent with this, the indole-3-acetic acid (IAA) level decreased significantly in *LsAP2*-OE plants (Fig. 5b). These findings indicate that LsAP2 may regulate leaf development through an auxin-related pathway.

Establishment of leaf adaxial–abaxial polarity is required for the flat outgrowth of the lamina^6^. Because the leaves of *LsAP2*-OE plants curled downwards, we speculated that LsAP2 might also affect adaxial–abaxial growth of the leaves. We specifically focused on the expression of genes related to adaxial–abaxial identity. The RNA-seq data showed that the *KAN2* and *YAB1* genes, which promote the abaxial fate of leaf primordia^10,11^, exhibited decreased expression levels in *LsAP2*-OE plants compared with WT (Fig. 5c). Further qRT-PCR analysis confirmed this result (Fig. 5d; Fig. S7b). Significantly, the expression levels of *LsKAN2* and *LsYAB1c* decreased by more than 10-fold and 5-fold in *LsAP2*-OE plants, respectively (Fig. 5d). These results imply that LsAP2 may also regulate abaxial identity-related genes.

### LsAP2 binds directly to *LsKAN2* and represses its expression

**Fig. 6:**
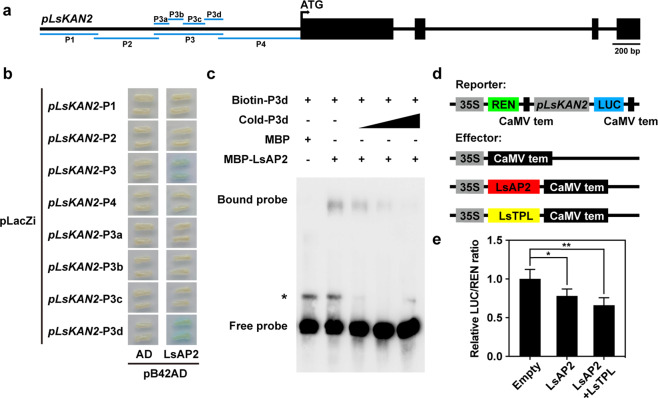
LsAP2 binds directly to *LsKAN2* and represses its expression. **a** Schematic representation of the *LsKAN2* gene structure used for LsAP2 binding assays. **b** Yeast one-hybrid assays show that LsAP2 binds directly to the *LsKAN2* promoter. Blue indicates an interaction. **c** Electrophoretic mobility shift assay (EMSA) showing that LsAP2 binds to the P3d fragment of the *LsKAN2* promoter. MBP maltose-binding protein. The asterisk indicates nonspecific binding. **d** Schematic representation of the reporter and effector constructs used in the dual-luciferase reporter assays. **e** Measurement of the relative LUC/REN ratio after transient coexpression of *pLsKAN2*:*LUC* with *35S:LsAP2* and *35S:LsTPL* in tobacco leaves. The pGreenII 62-SK empty vector was used as the control. The values are means ± SDs (*n* = 6). The data were normalized to a value of 1 for the control group. Significant differences were determined by Student’s *t* test (*P < 0.05; **P < 0.01)

To investigate whether LsAP2 regulated *LsKAN2* or *LsYAB1c* directly, we performed yeast one-hybrid assays to test the interactions between LsAP2 and the *LsKAN2* or *LsYAB1c* promoter. We found that LsAP2 bound to the P3 fragment of the *LsKAN2* promoter (Fig. 6b) but not to the promoter of *LsYAB1c* (Fig. S8), indicating that LsAP2 targeted *LsKAN2* directly. We also showed that LsAP2 bound to the P3d fragment of the *LsKAN2* promoter (Fig. 6b).

Furthermore, we performed an electrophoretic mobility shift assay (EMSA) to determine the interaction between LsAP2 and the *LsKAN2* promoter in vitro. Our results indicated that the MBP-LsAP2 protein bound to the P3d fragment of the *LsKAN2* promoter, whereas the maltose-binding protein (MBP) did not bind (Fig. 6c). To test whether LsAP2 can bind to the *LsKAN2* promoter in planta, we performed dual-luciferase reporter assays in tobacco leaves (Fig. 6d). Compared with the control group, the measured relative LUC/REN ratio decreased when the *35S:LsAP2* and *pLsKAN2:LUC* constructs were cotransformed into tobacco leaves (Fig. 6e), indicating that LsAP2 repressed the expression of *LsKAN2*. As expected, when we also transformed the LsTPL corepressor, the relative LUC/REN ratio decreased further (Fig. 6e). qRT-PCR analysis showed that *LsKAN2* was also expressed in leaves and shoot apexes (Fig. S9). Together, these results demonstrated that LsAP2 bound directly to *LsKAN2* and repressed its expression.

## Discussion

### Unique role of LsAP2 in regulating leaf development

AP2 belongs to the AP2/ERF superfamily and is well known for its role in floral development^27^. In addition, AP2 has numerous other roles in diverse plant species. For example, the rice (*Oryza sativa* L.) AP2 transcription factor SHATTERING ABORTION1 (SHAT1) controls seed shattering and seed size^41^. Five AP2 homologs have been identified in tomato, and SlAP2a acts as a major regulator of fruit ripening through regulating ethylene biosynthesis and signaling^42^. In wheat (*Triticum aestivum* L.), the *Q* gene encodes an AP2 transcription factor, and the Q protein interacts with the transcriptional corepressor TaTPL to control bread wheat spikelet architecture^43^. Previous studies have shown that *Arabidopsis AP2* is expressed in leaf primordia and young leaves^44,45^. However, whether AP2 regulates leaf development is largely unknown. In this study, we demonstrated that LsAP2 regulates leaf morphology in lettuce (Fig. 3), which is a newly discovered function of AP2 in the plant kingdom.

Our results showed that overexpression of *LsAP2* led to small and crinkly leaves (Fig. 3a, c). In severely affected *LsAP2*-OE plants, the leaves curled downwards, and many bulges were seen on the surface of the leaf blade (Fig. 3e). We found that knockout of *LsAP2* only led to an altered seed shape^26^, and there was no obvious change in leaf morphology between the WT and *LsAP2* knockout plants (Fig. 3a, c). Our results indicate that LsAP2 is a typical transcriptional repressor, implying that overexpression of *LsAP2* may result in a more distinct phenotype than that of the knockout plants. In addition, a whole-genome triplication event has been detected in lettuce^19^, and we previously confirmed that *LsAP2* had two paralogs in lettuce^26^. Therefore, the mild phenotype of *LsAP2* knockout plants may be explained by gene redundancy. This possibility suggests that a double or triple mutant of *LsAP2* and its paralogs will produce more dramatic phenotypes, which needs to be tested in future studies.

### LsAP2 may regulate leaf morphology by interacting with CIN-like TCPs

TCP proteins are plant-specific transcription factors and are involved in multiple developmental processes^12^. There are two classes of TCP transcription factors, class I and class II, and class II TCPs have been subclassified into two clades, namely, CIN-like and CYC/TB1. CIN-like TCPs are key regulators of leaf size and shape, and their important roles in leaf development have been shown to be conserved in many plants^33^. Recently, the lettuce CIN-like TCP transcription factor *LsTCP4* was identified as a candidate gene associated with leaf marginal serration and bolting time, and *LsTCP4* expression has been correlated with leaf shape^23^.

In this study, we showed that LsAP2 directly interacted with CIN-like TCPs and inhibited their transcriptional activation activity (Fig. 4; Fig. S5). Previous studies have shown that CIN-like TCPs promote the switch from cell proliferation to cell differentiation^13,15^. Therefore, the affected activity of CIN-like TCPs in *LsAP2*-OE plants may lead to abnormal cell proliferation and expansion (Fig. 3g), resulting in small leaves and excessive growth in the leaf interveinal regions (Fig. 3e). Furthermore, our RNA-seq data showed that many auxin-related genes were differentially expressed between WT and *LsAP2*-OE plants (Fig. 5a). It was shown that TCP transcription factors regulate cell differentiation by modulating auxin biosynthesis, transport, and response^14,46^. We speculate that LsAP2 may regulate leaf morphology by interacting with CIN-like TCPs and inhibiting their regulation of auxin-related genes. However, the direct target genes of CIN-like TCPs need to be identified in lettuce. On the other hand, LsAP2 may also regulate leaf development through an auxin-related pathway independent of CIN-like TCPs. In *LsAP2*-OE plants, auxin biosynthesis genes and activators of auxin signaling were downregulated, while genes encoding inhibitors of auxin signaling were upregulated (Fig. 5a). Auxin signaling plays crucial roles during leaf development^37,47^. The misregulation of auxin-related genes induced by LsAP2 may suppress the auxin response and signaling, thus affecting lettuce leaf development.

### LsAP2 may regulate leaf polarity by directly repressing *LsKAN2*

During leaf development, the adaxial side is adjacent to and the abaxial side is away from the shoot apical meristem^6^. In *Arabidopsis*, *AP2* transcripts were detected in developing leaves, especially on the adaxial sides of the leaf primordia^45^. However, whether AP2 promotes the adaxial domain or inhibits the abaxial domain of the leaf remains unknown. In this study, we showed that LsAP2 downregulated the expression of abaxial identity genes (Fig. 5c, d). LsAP2 also directly repressed *LsKAN2*, and the corepressor LsTPL enhanced the repression effect of LsAP2 on *LsKAN2* (Fig. 6).

Dorsoventrality in leaves depends on the precise expression pattern of adaxial–abaxial identity genes^48^. In lettuce, disrupted leaf dorsoventrality can result in leaf curvature and even leafy head^25^. Because overexpression of *LsAP2* repressed the abaxial identity gene *LsKAN2* (Fig. 5c, d), we speculate that the disrupted expression of *LsKAN2* may lead to excessive growth on the adaxial side of the leaf and suppress abaxial growth, resulting in leaves curled downwards (Fig. 3e). These results suggest that LsAP2 may regulate leaf polarity by directly repressing *LsKAN2*.

**Fig. 7:**
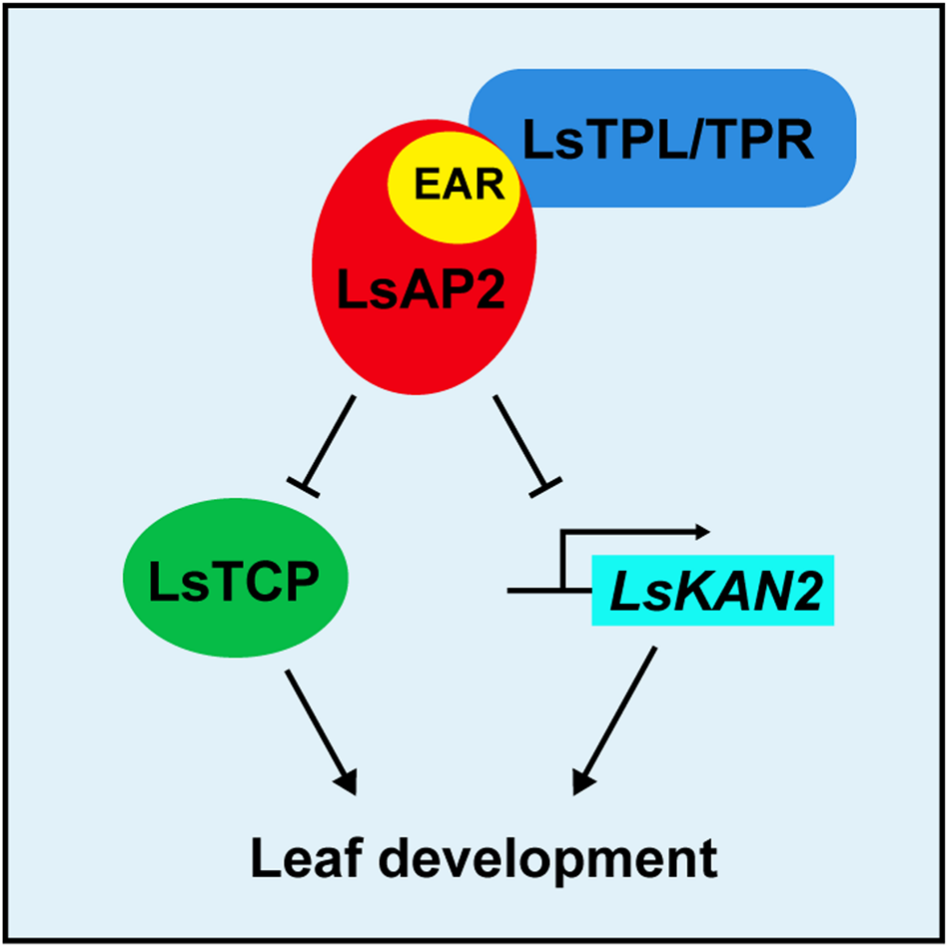
Proposed model for the function of LsAP2 during leaf development in lettuce. LsAP2 uses its EAR motif to recruit TPL/TPR corepressors and regulates lettuce leaf development via two pathways. LsAP2 may regulate leaf morphology by physically interacting with CIN-like TCPs and inhibiting their activity. LsAP2 may also regulate leaf polarity by directly binding to the promoter of *LsKAN2* and repressing its expression. Arrows, positive regulation; bars, negative regulation

In summary, our findings show that LsAP2 may regulate lettuce leaf development by inhibiting the activity of CIN-like TCPs and repressing the expression of *LsKAN2* (Fig. 7). These results provide insights into the regulatory mechanisms of leaf development in lettuce. Because leaf development requires fine-tuned coordination of cell proliferation and differentiation, a deeper understanding of the leaf development mechanisms of lettuce will contribute to the improvement of lettuce varieties in the future.

## Materials and methods

### Plant materials and growth conditions

The lettuce cultivar S39 was used in this study. The *pLsAP2:GUS* plants and the CRISPR/Cas9-mediated *LsAP2* knockout plants were obtained as described previously^26^. Briefly, to generate the *pLsAP2:GUS* construct, an ~2.4-kb long promoter region of *LsAP2* was cloned into the pBI121 vector to drive the *GUS* reporter gene. To generate the *LsAP2* knockout construct, two target sites were designed in the first exon of *LsAP2* and introduced into the pKSE401 vector. For GUS staining and phenotypic analysis, the plants were cultivated in growth chambers under a cycle of 16 h of light (200 μmol m^−2^ s^−1^) at 25 °C and 8 h of darkness at 18 °C.

### GUS staining analysis

The 5-, 10-, and 15-d-old seedlings of *pLsAP2:GUS* plants were used for the GUS staining analysis. GUS activity analysis was performed as described previously^49^. Briefly, different samples were immersed in GUS staining buffer and vacuum infiltrated for 15 min. The samples were stained overnight at 37 °C, washed with 70% ethanol a few times and immersed in fresh 70% ethanol before imaging. The samples were photographed using a stereomicroscope (Leica S8 APO, Germany) and a digital camera (Nikon D7000, Japan).

### RNA extraction and qRT-PCR

Total RNA from different lettuce tissues was extracted using a Quick RNA isolation Kit (Huayueyang, China). The cDNAs were synthesized using FastKing-RT SuperMix (Tiangen, China). qRT-PCR assays were performed using TB Green Premix Ex Taq II (Takara, Japan) in a real-time PCR system (QuantStudio 6 Flex; Applied Biosystems, USA). Three biological and three technical replicates were performed for each assay. *LsPP2A-1* and *LsTIP41* were used as internal reference genes to normalize the expression data^50^, and the 2^−ΔΔCT^ method was used to calculate the relative expression levels^51^. The primers used for the qRT-PCR assays are listed in Table S1.

### Lettuce transformation

To generate the *LsAP2*-OE construct, the full-length coding sequence (CDS) of *LsAP2* was cloned into the *Xba*I–*Sac*I sites of the pBI121 vector under the control of the cauliflower mosaic virus 35S (35S) promoter. The recombinant construct was transformed into *Agrobacterium* strain GV3101. The *LsAP2*-OE transgenic plants were obtained by *Agrobacterium*-mediated transformation of lettuce using a previously described method^26^. The primers used to generate the *LsAP2*-OE construct are listed in Table S1.

### Measurement of leaf area

One-month-old WT, *LsAP2*-OE, and *LsAP2* knockout plants were used to measure the leaf area, with 10 biological replicates. All the leaves from a plant were harvested and imaged using an Epson Perfection V800 Photo Scanner (Epson, Japan). The images were imported into WinRHIZO software (Regent Instruments Inc., Canada) for image analysis to calculate the leaf area.

### Scanning electron microscopy

The mature leaves of one-month-old WT and *LsAP2*-OE plants were used for scanning electron microscopy. Leaf samples were cut and fixed in formaldehyde-acetic acid-ethanol (FAA) buffer overnight. Then, the samples were dried at the critical point in liquid CO_2_. To obtain scanning electron micrographs of the leaf epidermal cells, the samples were gold plated and observed using a scanning electron microscope (Hitachi S-3400N, Japan).

### Subcellular localization

The full-length CDSs of *LsAP2* and *LsTCPs* without the stop codon were fused with GFP or mCherry by cloning into the *Hin*dIII–*Spe*I sites of the pSuper1300 vector (pCAMBIA1300 vector containing a Super promoter, which consists of three copies of the octopine synthase upstream activation sequence in front of the mannopine synthase promoter)^52^. The recombinant constructs were transformed into *Agrobacterium* strain GV3101 and infiltrated into tobacco leaves. After infiltration, the tobacco plants were grown under a 16-h light/8-h dark cycle for 2 days. After incubation, subcellular localization was detected using a confocal laser scanning microscope (Olympus FV3000, Japan). The primers used to generate the constructs are listed in Table S1.

### Dual-luciferase reporter assays

For transcriptional activity assays, the GAL4/UAS system was used to determine the transcriptional activity of the transcription factors^53^. The firefly luciferase (*LUC*) reporter gene was driven by the enhancer region of the 35S promoter, the upstream activation sequence (UAS) that was bound by the GAL4 protein, and a minimal 35S promoter (TATA). The *Renilla* luciferase (*REN*) reporter gene was driven by the 35S promoter in the same vector as an internal control. The VP16 transcriptional activation domain and full-length CDSs of *LsAP2* and *LsTCP3* were fused with the GAL4 DNA-binding domain (GAL4 DBD) and inserted into the *Bam*HI–*Hin*dIII sites of the pGreenII 62-SK vector as effectors. In addition, the full-length CDS of *LsAP2* without the stop codon was cloned into the pGAL4 DBD-VP16 vector to generate the GAL4 DBD-LsAP2-VP16 construct.

For the binding activity assays, the 1069-bp genomic fragment upstream of the *LsKAN2* start codon was cloned into the *Hin*dIII–*Bam*HI sites of the pGreenII 0800-LUC vector as the reporter. The *REN* reporter gene was driven by the 35S promoter in the same vector as an internal control. The full-length CDSs of *LsAP2* and *LsTPL* were cloned into the *Bam*HI–*Hin*dIII sites of the pGreenII 62-SK vector as effectors. The pGreenII 62-SK empty vector was used as the negative control.

Transient expression assays were performed as described previously^54^. Briefly, the recombinant constructs were transformed into *Agrobacterium* strain GV3101 (pSoup-P19) and infiltrated into tobacco leaves. After 2 days of incubation, LUC and REN activities were measured using a SpectraMax^®^ i3x Multi-Mode detection platform (Molecular Devices, USA) with a Dual-Luciferase Reporter Assay Kit (Promega, USA). The LUC to REN ratio was calculated as a measure of the transcriptional activity. The primers used to generate the constructs are listed in Table S1.

### Phylogenetic analysis

Lettuce TPL/TPR proteins and CIN-like TCP proteins were identified by BLASTP searches against the lettuce genome database using the amino acid sequences of annotated *Arabidopsis* proteins. Lettuce sequences were obtained from the CoGe database (https://genomevolution.org/coge/). *Arabidopsis* sequences were obtained from the TAIR database (https://www.arabidopsis.org/). *Antirrhinum* sequences were obtained from the snapdragon genome database (http://bioinfo.sibs.ac.cn/Am/). The obtained amino acid sequences were aligned using ClustalW^55^. Then, MEGA5 software was used to construct phylogenetic trees using the neighbor-joining method with 1000 bootstrap replicates^56^. The accession numbers of the amino acid sequences used to construct the phylogenetic trees are listed in Table S2.

### Yeast two-hybrid assays

The full-length CDS of *LsAP2* was cloned into the pGADT7 vector at the *Nde*I site for fusion with the GAL4 activation domain. Conserved leucine residues in the EAR motif of the LsAP2 protein were replaced by alanine residues by site-directed mutagenesis. To verify the interactions between LsAP2 and TPL/TPR proteins, the full-length CDS of *LsTPL* and the N-terminal sequences (618 bp) of *LsTPL* and *LsTPRs* were cloned into the pGBKT7 vector at the *Nde*I site for fusion with the GAL4 DBD. To test the interactions between LsAP2 and CIN-like TCPs in lettuce, the full-length CDSs of lettuce *CIN-like TCPs* were cloned into the pGBKT7 vector at the *Nde*I site for fusion with the GAL4 DBD. Approximately 0.1 µg of bait and prey plasmids were cotransformed into the yeast strain AH109 using the Matchmaker™ GAL4 Two-Hybrid System according to the manufacturer’s instructions (Clontech, USA). After growth at 28 °C for 3 days, yeast transformants were diluted and transferred to medium supplemented with SD/-Leu-Trp for growth of the yeast transformants or medium supplemented with SD/-Leu-Trp-His-Ade and 3-amino-1,2,4-triazole (3-AT) for protein interaction selection. The primers used to generate the constructs are listed in Table S1.

### Luciferase complementation imaging assays

The full-length CDSs of *LsAP2*, *LsAP2mEAR*, and *LsTCPs* without the stop codon were cloned into the *Kpn*I–*Sal*I sites of the pCAMBIA1300-nLUC vector for fusion with the N-terminus of the LUC fragment under the control of the 35S promoter. The N-terminal sequences were fused with the stop codon of *LsTPL* and *LsTPR1*, and the full-length CDSs of *LsAP2* and *LsTCPs* were cloned into the *Kpn*I–*Sal*I sites of the pCAMBIA1300-cLUC vector for fusion with the C-terminus of the LUC fragment under the control of the 35S promoter. LCI assays were performed as described previously^57^. Briefly, the constructs were transformed into *Agrobacterium* strain GV3101, which was then incubated at 28 °C overnight with shaking. The bacterial suspensions were adjusted to a final OD_600_ of 0.5 using infiltration buffer. Then, the bacterial suspensions with different construct combinations were infiltrated into tobacco leaves. After infiltration, the plants were grown under a 16-h light/8-h dark cycle for 2 days. Finally, the tobacco leaves were cut and sprayed with luciferin, and a chemiluminescence imaging apparatus (Roper Lumazone 1300B, USA) was used to capture the illumination signal. The primers used to generate the constructs are listed in Table S1.

### RNA-seq analysis

Young leaves of one-month-old WT and *LsAP2*-OE plants were used for RNA-seq, with three biological replicates. RNA-seq libraries were constructed and sequenced at Biomarker Technologies (BioMarker, China) using an Illumina NovaSeq 6000 platform. Analyses of the RNA-seq data were performed on the BMKCloud platform (http://www.biocloud.net). Briefly, the clean reads were aligned to the lettuce reference genome using HISAT2^58^. Differentially expressed genes (fold change ≥ 2, FDR < 0.01) were identified using the DESeq package^59^. KEGG enrichment analysis was performed using KOBAS^60^.

### Measurement of endogenous phytohormone

Approximately 0.5 g of young leaves of one-month-old WT and *LsAP2*-OE plants were harvested and used for measurement of endogenous phytohormones. Three biological replicates were performed for each sample. Extraction of endogenous phytohormones and quantification of auxin content by enzyme-linked immunosorbent assays were performed as described previously^26^.

### Yeast one-hybrid assays

The full-length CDS of *LsAP2* was cloned into the *Eco*RI–*Xho*I sites of the pB42AD vector. The promoter fragments of *LsKAN2* and *LsYAB1c* were cloned into the *Eco*RI–*Xho*I sites of the pLacZi vector to drive the *LacZ* reporter gene. Yeast one-hybrid assays were performed using a Matchmaker One-Hybrid System (Clontech, USA). Briefly, effector and reporter plasmids were cotransformed into yeast strain EGY48. Yeast transformants were grown on SD/-Ura-Trp plates for growth of the colonies, and then, the transformants were transferred to SD/Gal/Raf/-Ura-Trp plates containing X-gal for interaction selection. The primers used to generate the constructs are listed in Table S1.

### Electrophoretic mobility shift assays

The full-length CDS of *LsAP2* was cloned into the *Sal*I–*Pst*I sites of the pMal-c2X vector to generate the MBP-LsAP2 construct. The control plasmid and recombinant plasmid were transformed into *Escherichia coli* BL21 (DE3) to express the proteins. Proteins were purified using amylose resin (NEB, USA). Hot probes were synthesized and labeled with biotin at Shanghai Sangon Biotechnology (Sangon, China). EMSA was performed using a LightShift™ Chemiluminescent EMSA Kit (Thermo Fisher Scientific, USA) according to the manufacturer’s instructions. The primers used for the EMSA are listed in Table S1.

### Accession numbers

The accession numbers of the genes used in this study are listed in Table S2. RNA-seq data were deposited in the NCBI Gene Expression Omnibus database with the accession number GSE168886.

## Supplementary information

Figures S1-S9 and Tables S1, S2

Dataset S1

## Data Availability

The data that support the results are included in this article and its supplementary materials.
