# Supplementary material for: LsAP2 regulates leaf morphology by inhibiting CIN-like TCP transcription factors and repressing LsKAN2 in lettuce
Source: Hortic Res. 2021 Sep 1;8:184. doi: 10.1038/s41438-021-00622-y (PMC8408249; doi:10.1038/s41438-021-00622-y)
Supplement: Supplementary file 1 — Figures S1-S9 and Tables S1, S2 [file 41438_2021_622_MOESM1_ESM.docx]

**Supplementary Information**

**
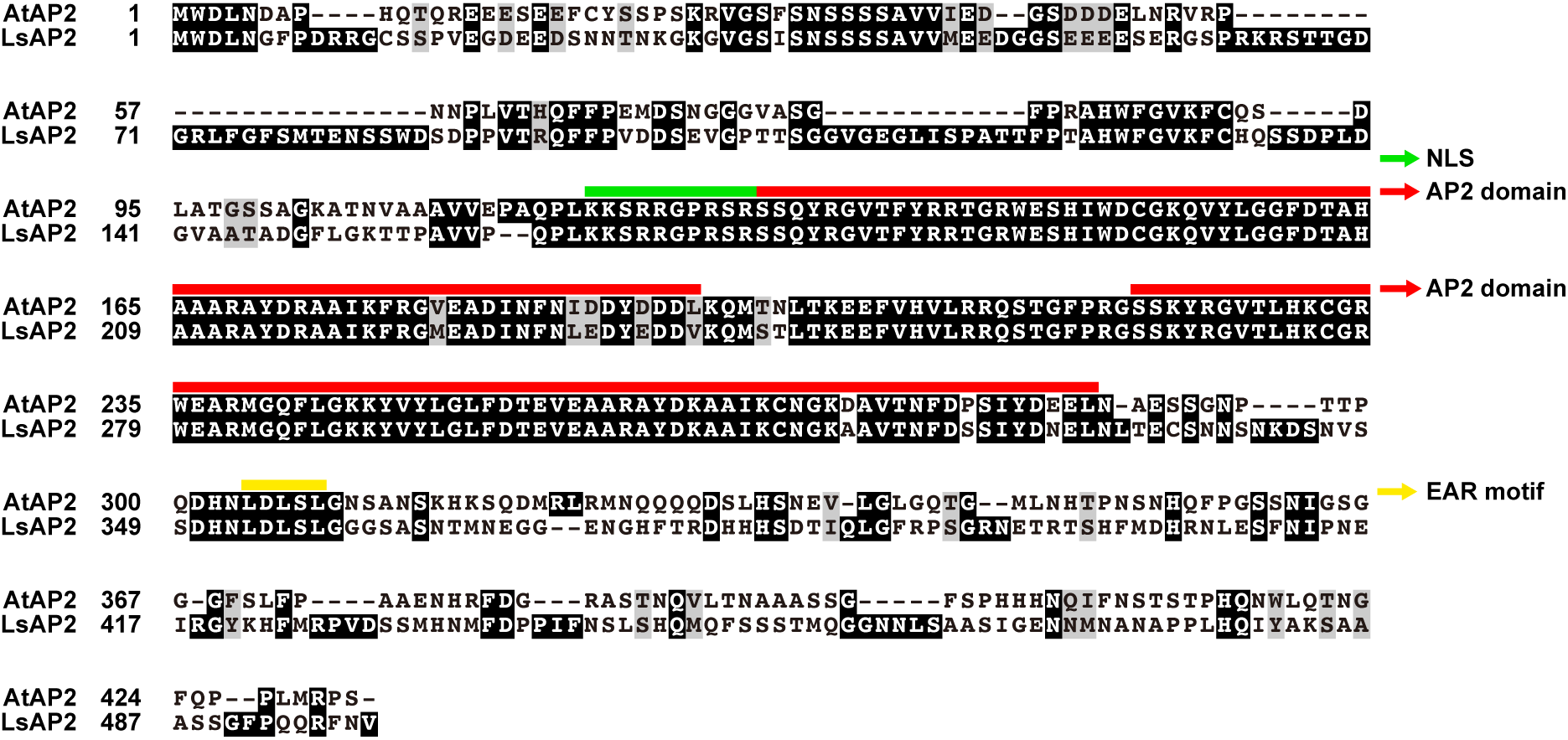
**

**Fig. S1. Alignment of the deduced amino acid sequences of AP2 proteins.**

AtAP2, *Arabidopsi*s AP2 sequence; LsAP2, lettuce AP2 sequence; NLS, nuclear localization sequence; EAR, ethylene-responsive element binding factor-associated amphiphilic repression.

**
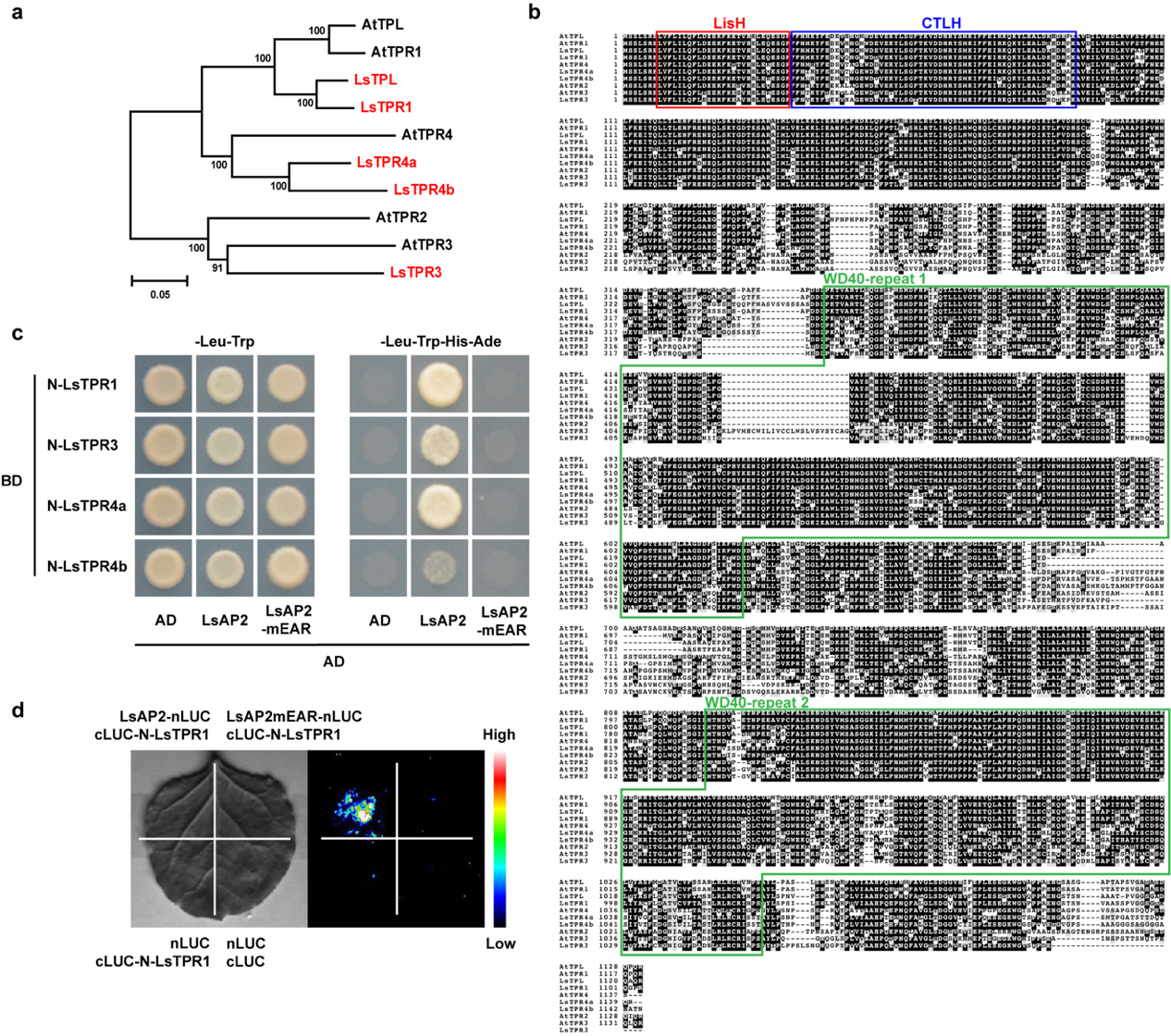
**

**Fig. S2. LsAP2 interacts with LsTPRs.**

(a) Phylogenetic analysis of the TPL/TPR proteins. Ls, *Lactuca sativa*; At, *Arabidopsis* *thaliana*. The lettuce TPL/TPRs are in red font. (b) Alignment of the deduced amino acid sequences of TPL/TPR proteins. Conserved domains are indicated by boxes. (c) Yeast two-hybrid assays show that LsAP2 interacts with LsTPRs through the EAR motif. AD, activation domain; BD, binding domain. (d) Luciferase complementation imaging (LCI) assays show the interaction between LsAP2 and N-LsTPR1.

**
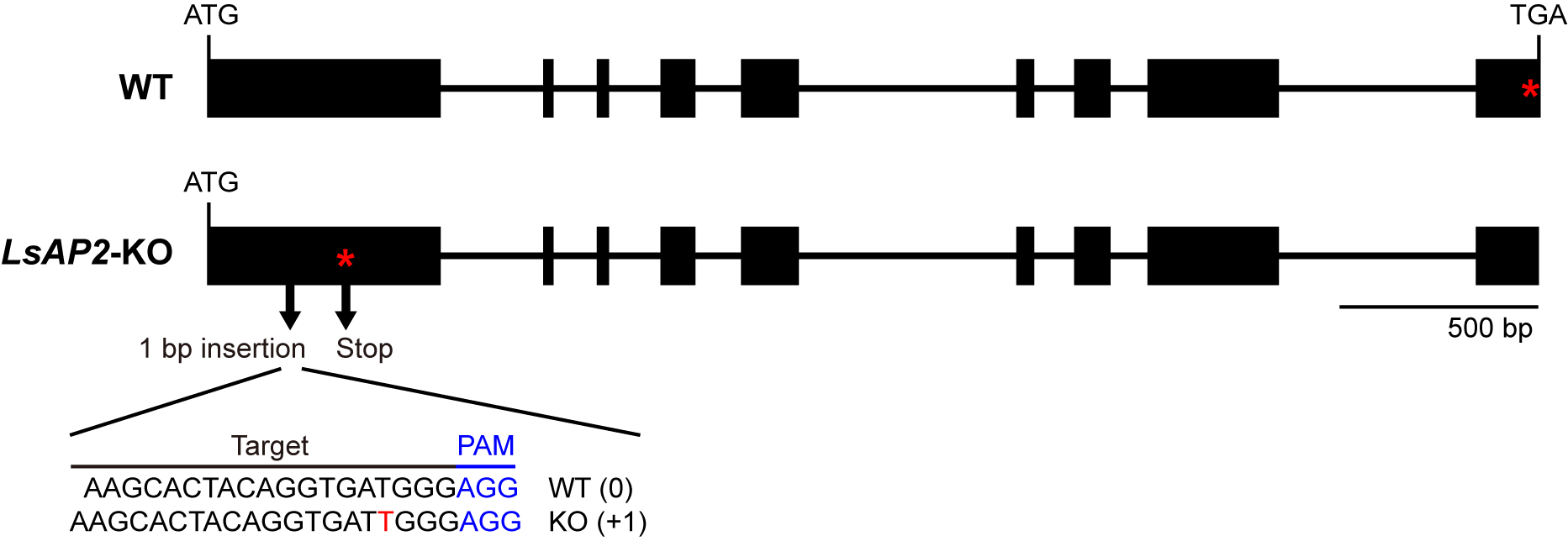
**

**Fig. S3.** **Schematic representation of the *LsAP2* knockout allele.**

The *LsAP2* knockout (KO) allele has a 1-bp insertion in the first exon compared with the wild type (WT), which causes a frameshift mutation and the introduction of a premature stop codon.

**
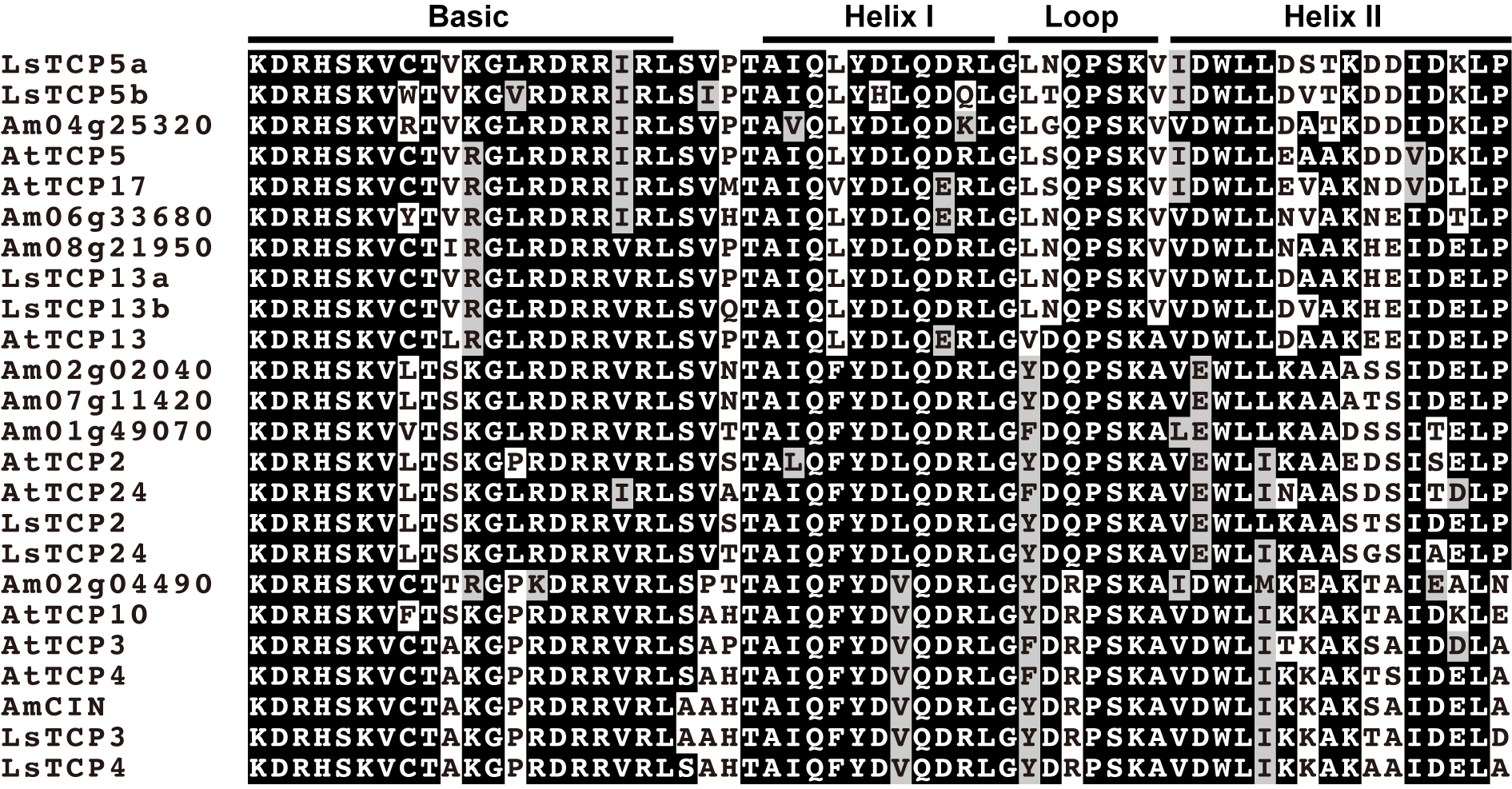
**

**Fig. S4.** **Alignment of the deduced amino acid sequences of the TCP domains from TCP proteins.**

The TCP proteins have a conserved basic helix–loop–helix (bHLH) motif called the TCP domain. Ls, *Lactuca sativa*; Am, *Antirrhinum* *majus*; At, *Arabidopsis* *thaliana*.

**
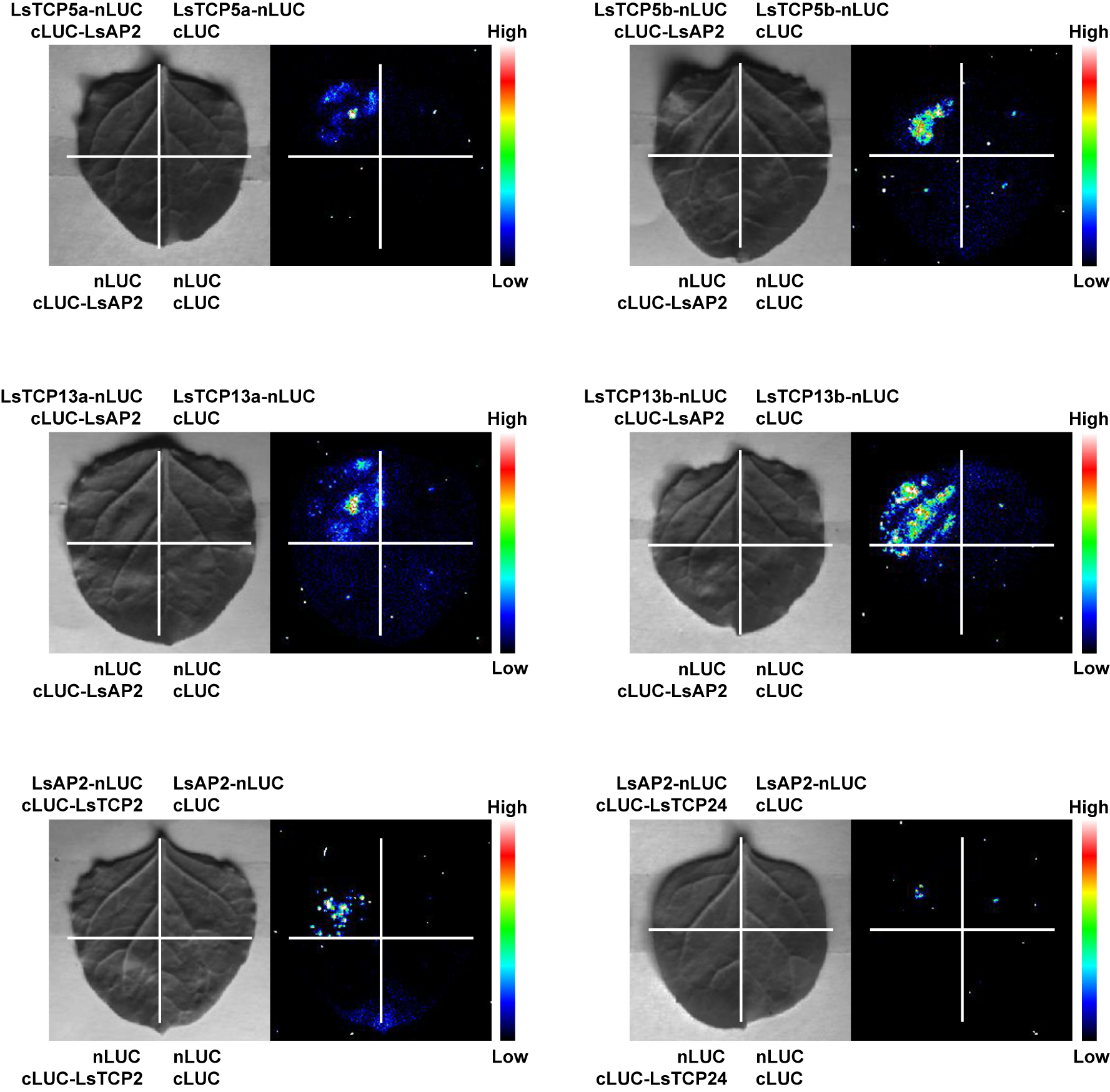
**

**Fig. S5. LCI assays show the interactions between LsAP2 and CIN-like TCPs.**

**
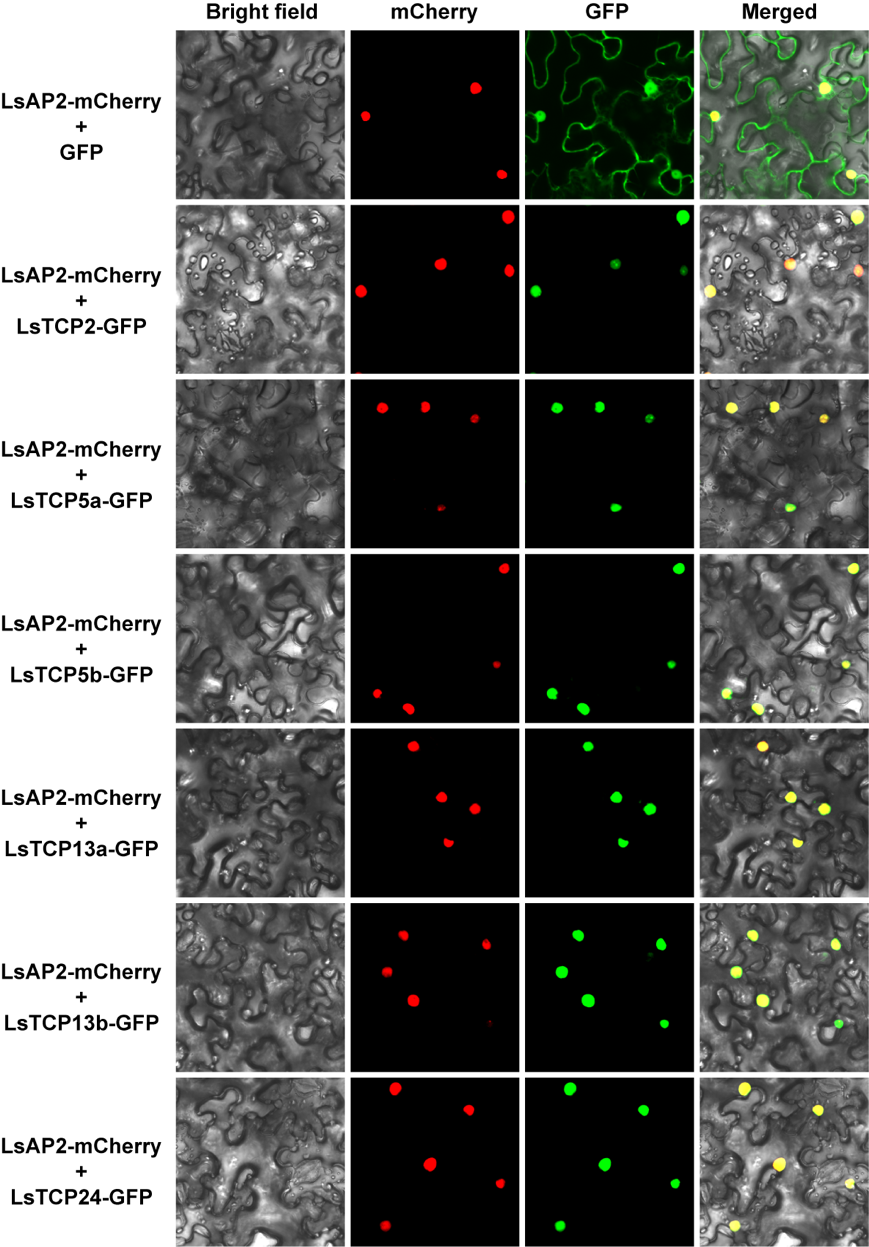
**

**Fig. S6. Subcellular localization of the CIN-like TCP proteins.**

**
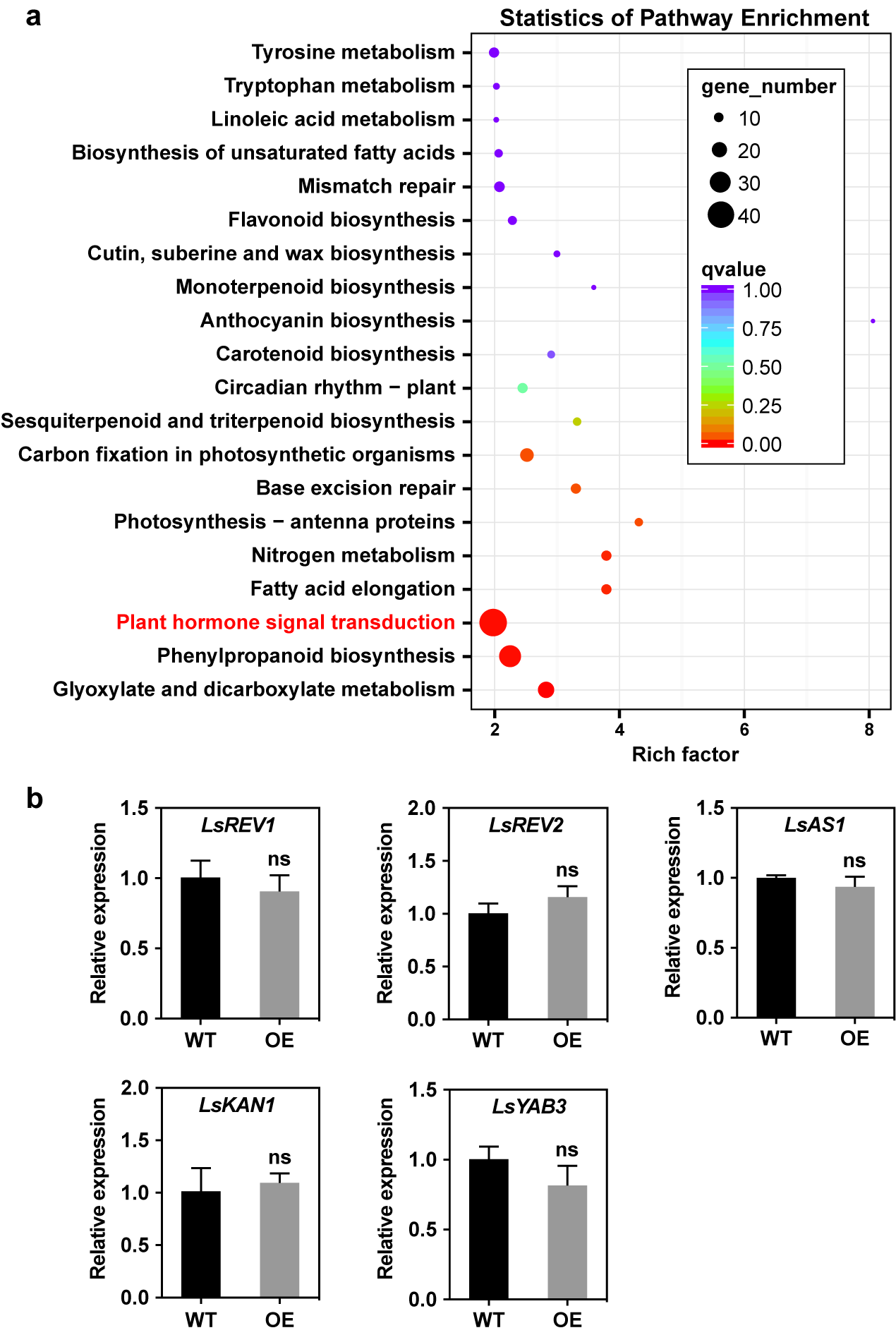
**

**Fig. S7. KEGG enrichment analysis of the differentially expressed genes and expression analysis of polarity-related genes.**

(a) KEGG enrichment analysis of the differentially expressed genes. (b) The qRT-PCR analysis shows the expression levels of adaxial–abaxial identity genes between WT and *LsAP2*-OE plants. Values are means ± SD (*n* = 3). Data were normalized as 1 in WT. Significant differences were determined by the Student’s *t* test (ns, not significant).

**
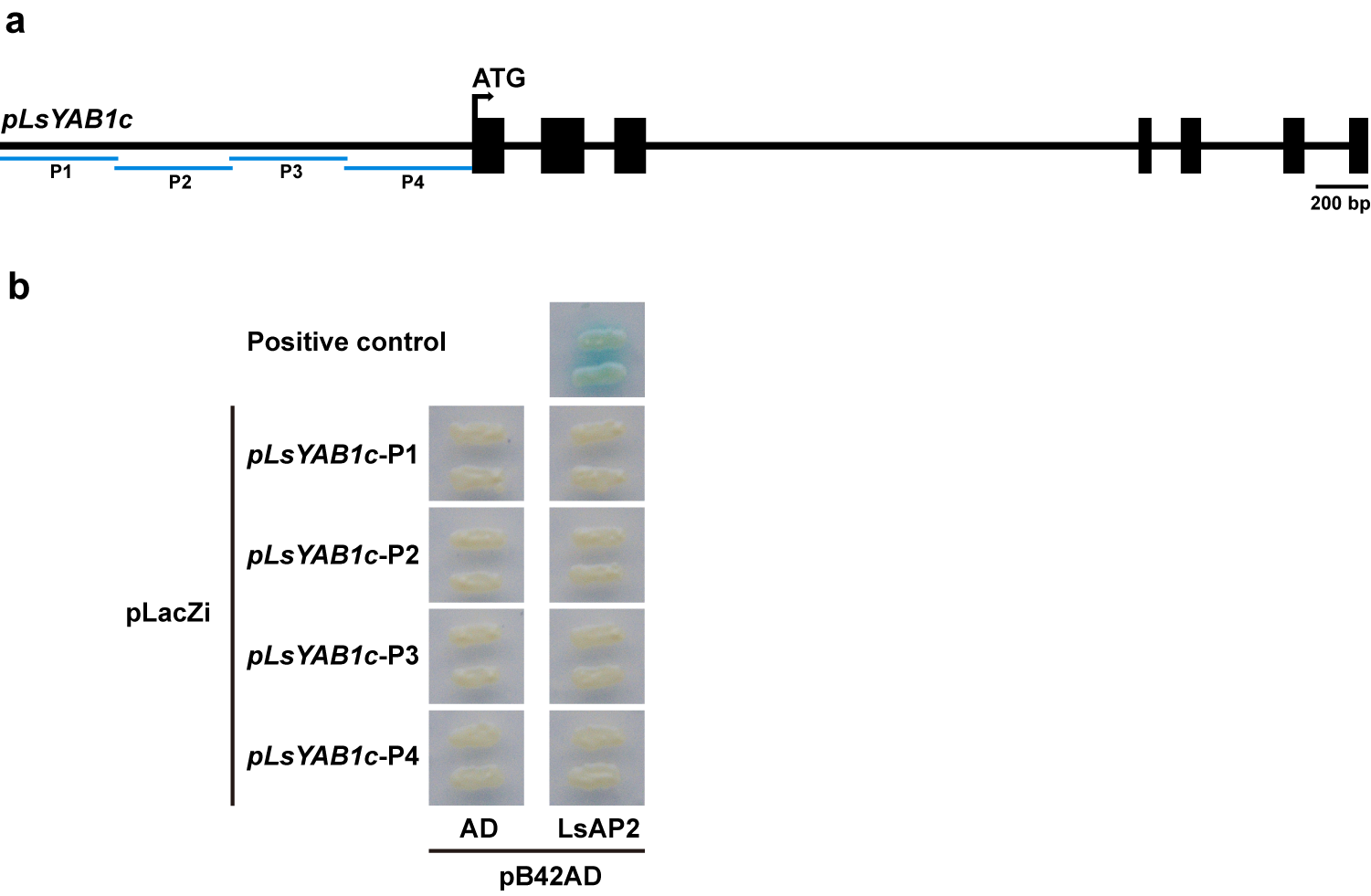
**

**Fig. S8.** **Interaction analysis between LsAP2 and *LsYAB1c* promoter.**

(a) Schematic representation of the *LsYAB1c* gene structure used for the LsAP2 binding assays. (b) Yeast one-hybrid assays test the interactions between LsAP2 and *LsYAB1c* promoter. Blue indicates an interaction.

**
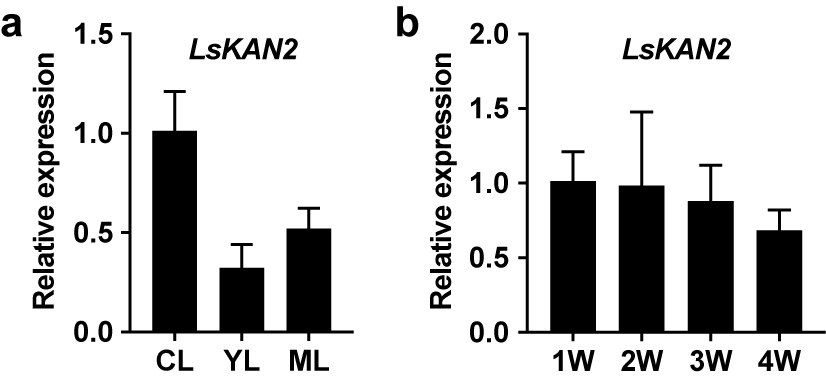
**

**Fig. S9. Expression of *LsKAN2* in leaves and** **shoot apexes.**

(a) Expression of *LsKAN2* in different leaves. CL, cotyledon; YL, young leaf; ML, mature leaf. Values are means ± SD (*n* = 3). Data were normalized as 1 in young leaf. (b) Expression of *LsKAN2* in shoot apexes at different vegetative growth stages. 1W, 1 week; 2W, 2 weeks, 3W, 3 weeks; 4W, 4 weeks. Values are means ± SD (*n* = 3). Data were normalized as 1 in 1-week-old shoot apex.

**Table S1. Primers used in this study.**

| **Purpose** | **Primer sequence (5′−3′)** |
| --- | --- |
| **qRT-PCR** | |
| LsAP2-qRT-F | GTGGTGGCTCGGCTTCAAATA |
| LsAP2-qRT-R | GGTCCATAAAATGCGATGTCCTT |
| LsPP2A-1-qRT-F | ATTCATGGTCAATTCTACGATCTGGT |
| LsPP2A-1-qRT-R | GAATAATACCCGCGATCAACATAATC |
| LsTIP41-qRT-F | TTTGTATGGAGATGAATTGGCTGATA |
| LsTIP41-qRT-R | CGTAAGAGAAGAAACCAACAGCTAGG |
| LsTCP2-qRT-F | TTACCGTCGTCTCTTTTACCTC |
| LsTCP2-qRT-R | GATGAAGAAGGGCAGATTTGTG |
| LsTCP3-qRT-F | TCGCCGCCAACACAAACAAC |
| LsTCP3-qRT-R | ATAGAAGACGGGTGGTCAACTG |
| LsTCP4-qRT-F | ATTCAACAATTCTCAGAGGGGA |
| LsTCP4-qRT-R | GAAGATGGGTGATGGAAAGCTA |
| LsTCP5a-qRT-F | CTTCAGAAGGAAAAGACGTTCG |
| LsTCP5a-qRT-R | TTAAAACCTGCCCATTGTCTTG |
| LsTCP5b-qRT-F | TCAGCAGTTTTCGAACCTATCT |
| LsTCP5b-qRT-R | CAAATGGCATTACGTAAGGAGG |
| LsTCP13a-qRT-F | CACAGTCCTATTTCCCTTCACACA |
| LsTCP13a-qRT-R | GTCGCATCCCTTGGTTTATTG |
| LsTCP13b-qRT-F | CATCTTTACCAGTTGTGTGCAA |
| LsTCP13b-qRT-R | GCTTCCGAACTGTGATAATGAC |
| LsTCP24-qRT-F | ATTGAACGACAGGTGAGTAAGT |
| LsTCP24-qRT-R | CGATGCTCATGGTTTCGTAAAT |
| LsREV1-qRT-F | GCATTGGCGACAACACATC |
| LsREV1-qRT-R | ACACTTCTGACATACTGGCG |
| LsREV2-qRT-F | CAAAATGTCCCTCCAGCAGT |
| LsREV2-qRT-R | TGTGAATCTTGTGGGTCGCA |
| LsAS1-qRT-F | TTCACTCCGACCATCCACTCA |
| LsAS1-qRT-R | TGCTTTGCTTCCTCCACCTC |
| LsKAN1-qRT-F | CAATTTGTCCTTGTAACGGACA |
| LsKAN1-qRT-R | CACAATCCAAAGAAGTCAAGGG |
| LsKAN2-qRT-F | AATCAGTCCACCCACCAGCA |
| LsKAN2-qRT-R | CGACAAGGAAAGCCCGAAAG |
| LsYAB1a-qRT-F | TCAACCCGTGAAGAAACCCA |
| LsYAB1a-qRT-R | TCAGTAAGGCGAAACACCCAC |
| LsYAB1b-qRT-F | AAACAAACATGTCATCATCGGC |
| LsYAB1b-qRT-R | CACAAAAGTTGCACTGGACATA |
| LsYAB1c-qRT-F | CTGCTTGCGGAGGATTTAGG |
| LsYAB1c-qRT-R | GATTGTAGGCGGAGGGGACT |
| LsYAB3-qRT-F | ACCGTCCTCGCTGTTAGTGTT |
| LsYAB3-qRT-R | TCCTGGGGCAGTTGTAGAAGTA |
| **Lettuce transformation** | |
| LsAP2-pBI121-F | ACGGGGGACTCTAGAATGTGGGATCTAAACGGGTTTC |
| LsAP2-pBI121-R | GGGAAATTCGAGCTCTCAAACATTGAATCTTTGCTGT |
| **Subcellular localization** | |
| LsAP2-pSuper1300-F | GACTCTAGAAAGCTTATGTGGGATCTAAACGGGTTTC |
| LsAP2-pSuper1300-R | ACCGGATCCACTAGTAACATTGAATCTTTGCTGT |
| LsTCP2-pSuper1300-F | GACTCTAGAAAGCTTATGGAGGTGGATGAAATTCAA |
| LsTCP2-pSuper1300-R | ACCGGATCCACTAGTGTTCTTTGTTTTTTCTTTGTG |
| LsTCP3-pSuper1300-F | GACTCTAGAAAGCTTATGGGAGGAAGTTATCAGTACGA |
| LsTCP3-pSuper1300-R | ACCGGATCCACTAGTATGGCGAGAATCAGAGGAAG |
| LsTCP4-pSuper1300-F | GACTCTAGAAAGCTTATGGGAGAAAGTTATCAGTATCAC |
| LsTCP4-pSuper1300-R | ACCGGATCCACTAGTATGGCGAGAATCGGAGGATG |
| LsTCP5a-pSuper1300-F | GACTCTAGAAAGCTTATGTTCAACAATACTTCAGAAGGA |
| LsTCP5a-pSuper1300-R | ACCGGATCCACTAGTGCTATCAACATTCTTCTGTGA |
| LsTCP5b-pSuper1300-F | GACTCTAGAAAGCTTATGTTTTCCAGATTAAGGATTCAA |
| LsTCP5b-pSuper1300-R | ACCGGATCCACTAGTACCATTGTTATTATTGCTTTG |
| LsTCP13a-pSuper1300-F | GACTCTAGAAAGCTTATGAATATTTCAAATTTAAGAGAA |
| LsTCP13a-pSuper1300-R | ACCGGATCCACTAGTTTTGTGTGGTTCATGCCCAC |
| LsTCP13b-pSuper1300-F | GACTCTAGAAAGCTTATGAATATCTTGAATACAAGAGAA |
| LsTCP13b-pSuper1300-R | ACCGGATCCACTAGTTGAATGTCCATTTTCTTCAGA |
| LsTCP24-pSuper1300-F | GACTCTAGAAAGCTTATGGAGGTGGATGAAATTGAACG |
| LsTCP24-pSuper1300-R | ACCGGATCCACTAGTGTTCTTTGACTTTTCCTTGTG |
| **Dual-luciferase reporter assays** | |
| GAL4 DBD-VP16-F | GAGGAGGACCTGCATATGGCCCCCCCGACCGATGTCA |
| GAL4 DBD-VP16-R | GGCCTCCATGGCCATATGCTACCCACCGTACTCGTCAA |
| GAL4 DBD-LsAP2-F | GAGGAGGACCTGCATATGATGTGGGATCTAAACGGGTTTC |
| GAL4 DBD-LsAP2-R | GGCCTCCATGGCCATATGTCAAACATTGAATCTTTGCTGT |
| GAL4 DBD-LsTCP3-F | GAGGAGGACCTGCATATGATGGGAGGAAGTTATCAGTACGA |
| GAL4 DBD-LsTCP3-R | GGCCTCCATGGCCATATGTCAATGGCGAGAATCAGAGGA |
| GAL4 DBD-62-SK-F | TCTAGAACTAGTGGATCCATGAAGCTACTGTCTTCTATCGA |
| GAL4 DBD-62-SK-R | GACGGTATCGATAAGCTTTTACGATACAGTCAACTGTCTTTG |
| GAL4 DBD-VP16-62-SK-R | GACGGTATCGATAAGCTTCTACCCACCGTACTCGTCAA |
| GAL4 DBD-LsAP2-62-SK-R | GACGGTATCGATAAGCTTTCAAACATTGAATCTTTGCTGT |
| GAL4 DBD-LsTCP3-62-SK-R | GACGGTATCGATAAGCTTTCAATGGCGAGAATCAGAGGA |
| GAL4 DBD-LsAP2-VP16-62-SK-F | CAGAGGAGGACCTGCATATGATGTGGGATCTAAACGGGTTTC |
| GAL4 DBD-LsAP2-VP16-62-SK-R | TCGGTCGGGGGGGCCATATGAACATTGAATCTTTGCTGT |
| LsAP2-62-SK-F | TCTAGAACTAGTGGATCCATGTGGGATCTAAACGGGTTTC |
| LsAP2-62-SK-R | GACGGTATCGATAAGCTTTCAAACATTGAATCTTTGCTGT |
| LsTPL-62-SK-F | TCTAGAACTAGTGGATCCATGTCGTCTCTAAGTAGGGAGC |
| LsTPL-62-SK-R | GACGGTATCGATAAGCTTTCATCTTTGAGCTTGATCCGC |
| pLsKAN2-0800-LUC-F | GGTATCGATAAGCTTCTCATAGATCATAGATGTATGTG |
| pLsKAN2-0800-LUC-R | AGAACTAGTGGATCCTTCAGCTACCAAGTTCTCTACTT |
| **Yeast two-hybrid assays** | |
| LsAP2-AD-F | GATTACGCTCATATGATGTGGGATCTAAACGGGTTTC |
| LsAP2-AD-R | CTCCATGGCCATATGTCAAACATTGAATCTTTGCTGT |
| LsAP2mEAR-F | gcTGATgcGAGCgcAGGTGGTGGCTCGGCTTCA |
| LsAP2mEAR-R | TgcGCTCgcATCAgcATTATGGTCCGATGATAC |
| LsTPL-BD-F | GAGGAGGACCTGCATATGATGTCGTCTCTAAGTAGGGAG |
| LsTPL-BD-R | GGCCTCCATGGCCATATGTCATCTTTGAGCTTGATCCGC |
| N-LsTPL-BD-F | GAGGACCTGCATATGATGTCGTCTCTAAGTAGGGAGCTT |
| N-LsTPL-BD-R | CTCCATGGCCATATGTCATTGACCACATGAATGGTCAACAAACAAA |
| N-LsTPR1-BD-F | GAGGACCTGCATATGATGTCGTCTCTAAGTAGAGAGCTC |
| N-LsTPR1-BD-R | CTCCATGGCCATATGTCATTGTCCACATGAATGATCCACAAATAAG |
| N-LsTPR3-BD-F | GAGGACCTGCATATGATGTCGTCACTGAGTAGAGAATTGG |
| N-LsTPR3-BD-R | CTCCATGGCCATATGTCAAGGTGTGCATGAGTGGTCTATG |
| N-LsTPR4a-BD-F | GAGGACCTGCATATGATGTCGTCCCTTAGTAGAGAGCT |
| N-LsTPR4a-BD-R | CTCCATGGCCATATGTCATTGACCACAAGAATGGTCAACAAAC |
| N-LsTPR4b-BD-F | GAGGACCTGCATATGATGTCGTCTCTCAGCAGAGAGC |
| N-LsTPR4b-BD-R | CTCCATGGCCATATGTCATTGACCACAAGAATGATCAATAAAAAGAGT |
| LsTCP2-BD-F | GAGGACCTGCATATGATGGAGGTGGATGAAATTCAAAGAC |
| LsTCP2-BD-R | CTCCATGGCCATATGTCAGTTCTTTGTTTTTTCTTTGTGATGATC |
| LsTCP3-BD-F | GAGGACCTGCATATGATGGGAGGAAGTTATCAGTACGA |
| LsTCP3-BD-R | CTCCATGGCCATATGTCAATGGCGAGAATCAGAGGA |
| LsTCP4-BD-F | GAGGACCTGCATATGATGGGAGAAAGTTATCAGTATCACAAC |
| LsTCP4-BD-R | CTCCATGGCCATATGTCAATGGCGAGAATCGGAGG |
| LsTCP5a-BD-F | GAGGACCTGCATATGATGTTCAACAATACTTCAGAAGGAAAAG |
| LsTCP5a-BD-R | CTCCATGGCCATATGTCAGCTATCAACATTCTTCTGTGAAC |
| LsTCP5b-BD-F | GAGGACCTGCATATGATGTTTTCCAGATTAAGGATTCAATC |
| LsTCP5b-BD-R | CTCCATGGCCATATGTCAACCATTGTTATTATTGCTTTGTG |
| LsTCP13a-BD-F | GAGGACCTGCATATGATGAATATTTCAAATTTAAGAGAATCAAACCAC |
| LsTCP13a-BD-R | CTCCATGGCCATATGTCATTTGTGTGGTTCATGCCC |
| LsTCP13b-BD-F | GAGGACCTGCATATGATGAATATCTTGAATACAAGAGAATCCG |
| LsTCP13b-BD-R | CTCCATGGCCATATGTCATGAATGTCCATTTTCTTCAGAAG |
| LsTCP24-BD-F | GAGGACCTGCATATGATGGAGGTGGATGAAATTGAACG |
| LsTCP24-BD-R | CTCCATGGCCATATGTTAGTTCTTTGACTTTTCCTTGTGATGATC |
| **Luciferase complementation imaging assays** | |
| LsAP2-nLuc-F | GACGAGCTCGGTACCATGTGGGATCTAAACGGGTTTC |
| LsAP2-nLuc-R | CGAGATCTGGTCGACAACATTGAATCTTTGCTGT |
| LsAP2-cLuc-F | TCCCGGGGCGGTACCATGTGGGATCTAAACGGGTTTC |
| LsAP2-cLuc-R | GCTCTGCAGGTCGACTCAAACATTGAATCTTTGCTGT |
| N-LsTPL-cLuc-F | TCCCGGGGCGGTACCATGTCGTCTCTAAGTAGGGAGC |
| N-LsTPL-cLuc-R | GCTCTGCAGGTCGACTCATTGACCACATGAATGGTCAA |
| N-LsTPR1-cLuc-F | TCCCGGGGCGGTACCATGTCGTCTCTAAGTAGAGAGCTC |
| N-LsTPR1-cLuc-R | GCTCTGCAGGTCGACTCATTGTCCACATGAATGATCCACA |
| LsTCP2-cLuc-F | TCCCGGGGCGGTACCATGGAGGTGGATGAAATTCAAAGA |
| LsTCP2-cLuc-R | GCTCTGCAGGTCGACTCAGTTCTTTGTTTTTTCTTTGTG |
| LsTCP3-cLuc-F | TCCCGGGGCGGTACCATGGGAGGAAGTTATCAGTACGA |
| LsTCP3-cLuc-R | GCTCTGCAGGTCGACTCAATGGCGAGAATCAGAGGA |
| LsTCP4-cLuc-F | TCCCGGGGCGGTACCATGGGAGAAAGTTATCAGTATCAC |
| LsTCP4-cLuc-R | GCTCTGCAGGTCGACTCAATGGCGAGAATCGGAGG |
| LsTCP24-cLuc-F | TCCCGGGGCGGTACCATGGAGGTGGATGAAATTGAACG |
| LsTCP24-cLuc-R | GCTCTGCAGGTCGACTTAGTTCTTTGACTTTTCCTTGTG |
| LsTCP5a-nLuc-F | GACGAGCTCGGTACCATGTTCAACAATACTTCAGAAGGA |
| LsTCP5a-nLuc-R | CGAGATCTGGTCGACGCTATCAACATTCTTCTGTGA |
| LsTCP5b-nLuc-F | GACGAGCTCGGTACCATGTTTTCCAGATTAAGGATTCAA |
| LsTCP5b-nLuc-R | CGAGATCTGGTCGACACCATTGTTATTATTGCTTTG |
| LsTCP13a-nLuc-F | GACGAGCTCGGTACCATGAATATTTCAAATTTAAGAGAA |
| LsTCP13a-nLuc-R | CGAGATCTGGTCGACTTTGTGTGGTTCATGCCCAC |
| LsTCP13b-nLuc-F | GACGAGCTCGGTACCATGAATATCTTGAATACAAGAGAA |
| LsTCP13b-nLuc-R | CGAGATCTGGTCGACTGAATGTCCATTTTCTTCAGA |
| **Yeast one-hybrid assays** | |
| LsAP2-pB42AD-F | GCCTCTCCCGAATTCATGTGGGATCTAAACGGGTTTC |
| LsAP2-pB42AD-R | CCAAAGCTTCTCGAGTCAAACATTGAATCTTTGCTGT |
| pLsKAN2-P1-pLacZi-F | TATTGGATCGAATTCCTCACTCTTCGATAAGAGATATG |
| pLsKAN2-P1-pLacZi-R | AGCACATGCCTCGAGGTCACTTTTGCTTTTCATGT |
| pLsKAN2-P2-pLacZi-F | TATTGGATCGAATTCACATGAAAAGCAAAAGTGAC |
| pLsKAN2-P2-pLacZi-R | AGCACATGCCTCGAGCACATACATCTATGATCTATGAG |
| pLsKAN2-P3-pLacZi-F | TATTGGATCGAATTCCTCATAGATCATAGATGTATGTG |
| pLsKAN2-P3-pLacZi-R | AGCACATGCCTCGAGGCTTTCTTTTATGTGGACGTG |
| pLsKAN2-P4-pLacZi-F | TATTGGATCGAATTCCACGTCCACATAAAAGAAAGC |
| pLsKAN2-P4-pLacZi-R | AGCACATGCCTCGAGTTCAGCTACCAAGTTCTCTACTT |
| pLsKAN2-P3a-pLacZi-F | TATTGGATCGAATTCCTCATAGATCATAGATGTATGTG |
| pLsKAN2-P3a-pLacZi-R | AGCACATGCCTCGAGCTTTTGTAATCTGACCACAATG |
| pLsKAN2-P3b-pLacZi-F | TATTGGATCGAATTCCATTGTGGTCAGATTACAAAAG |
| pLsKAN2-P3b-pLacZi-R | AGCACATGCCTCGAGGCACTTTATCTTTTTCATGATTTG |
| pLsKAN2-P3c-pLacZi-F | TATTGGATCGAATTCCAAATCATGAAAAAGATAAAGTGC |
| pLsKAN2-P3c-pLacZi-R | AGCACATGCCTCGAGCTTTACCATCTTTTCCTTTTCTTC |
| pLsKAN2-P3d-pLacZi-F | TATTGGATCGAATTCGAAGAAAAGGAAAAGATGGTAAAG |
| pLsKAN2-P3d-pLacZi-R | AGCACATGCCTCGAGGCTTTCTTTTATGTGGACGTG |
| pYAB1c-P1-pLacZi-F | TATTGGATCGAATTCGGAGTAGACTTAAAACAAAGAACT |
| pYAB1c-P1-pLacZi-R | AGCACATGCCTCGAGCATTCTTGCTCCCTTCCCAAG |
| pYAB1c-P2-pLacZi-F | TATTGGATCGAATTCCTTGGGAAGGGAGCAAGAATG |
| pYAB1c-P2-pLacZi-R | AGCACATGCCTCGAGGATGGTACGTAAATTGTAAGGC |
| pYAB1c-P3-pLacZi-F | TATTGGATCGAATTCGCCTTACAATTTACGTACCATC |
| pYAB1c-P3-pLacZi-R | AGCACATGCCTCGAGATAGTCTAGCTAGGGAGCTC |
| pYAB1c-P4-pLacZi-F | TATTGGATCGAATTCGAGCTCCCTAGCTAGACTAT |
| pYAB1c-P4-pLacZi-R | AGCACATGCCTCGAGGGTTAGTTTGTTTGGAGATTTATG |
| **Electrophoretic mobility shift assays** | |
| LsAP2-pMal-c2X-F | TCCTCTAGAGTCGACATGTGGGATCTAAACGGGTTTC |
| LsAP2-pMal-c2X-R | CAAGCTTGCCTGCAGTCAAACATTGAATCTTTGCTGT |
| pLsKAN2-P3d-F | TTTACCTTTTTTTATTTTTATTTTTATTTTTTTCCTTTCTCATCTTTTTTTTCTTCCTTTCTTCTTTCC |
| pLsKAN2-P3d-R | GGAAAGAAGAAAGGAAGAAAAAAAAGATGAGAAAGGAAAAAAATAAAAATAAAAATAAAAAAAGGTAAA |
| pLsKAN2-P3d-bio-F | bio_TTTACCTTTTTTTATTTTTATTTTTATTTTTTTCCTTTCTCATCTTTTTTTTCTTCCTTTCTTCTTTCC |
| pLsKAN2-P3d-bio-R | bio_GGAAAGAAGAAAGGAAGAAAAAAAAGATGAGAAAGGAAAAAAATAAAAATAAAAATAAAAAAAGGTAAA |

**Table S2.** **Accession numbers of the genes used in this study.**

| **Species** | **Gene name** | **Accession number** |
| --- | --- | --- |
| *Lactuca sativa* | *LsAP2* | Lsat_1_v5_gn_3_89681 |
| *Lactuca sativa* | *LsPP2A-1* | Lsat_1_v5_gn_8_160621 |
| *Lactuca sativa* | *LsTIP41* | Lsat_1_v5_gn_5_116421 |
| *Lactuca sativa* | *LsTPL* | Lsat_1_v5_gn_5_8840 |
| *Lactuca sativa* | *LsTPR1* | Lsat_1_v5_gn_1_31280 |
| *Lactuca sativa* | *LsTPR3* | Lsat_1_v5_gn_7_73160 |
| *Lactuca sativa* | *LsTPR4a* | Lsat_1_v5_gn_5_96921 |
| *Lactuca sativa* | *LsTPR4b* | Lsat_1_v5_gn_8_62341 |
| *Lactuca sativa* | *LsTCP2* | Lsat_1_v5_gn_4_29640 |
| *Lactuca sativa* | *LsTCP3* | Lsat_1_v5_gn_9_99020 |
| *Lactuca sativa* | *LsTCP4* | Lsat_1_v5_gn_5_127021 |
| *Lactuca sativa* | *LsTCP5a* | Lsat_1_v5_gn_4_110881 |
| *Lactuca sativa* | *LsTCP5b* | Lsat_1_v5_gn_5_22620 |
| *Lactuca sativa* | *LsTCP13a* | Lsat_1_v5_gn_3_97300 |
| *Lactuca sativa* | *LsTCP13b* | Lsat_1_v5_gn_4_171960 |
| *Lactuca sativa* | *LsTCP24* | Lsat_1_v5_gn_7_27781 |
| *Lactuca sativa* | *LsYUC4a* | Lsat_1_v5_gn_1_42980 |
| *Lactuca sativa* | *LsYUC4b* | Lsat_1_v5_gn_3_83960 |
| *Lactuca sativa* | *LsYUC6* | Lsat_1_v5_gn_1_48821 |
| *Lactuca sativa* | *LsIAA3* | Lsat_1_v5_gn_8_91920 |
| *Lactuca sativa* | *LsIAA14* | Lsat_1_v5_gn_3_125741 |
| *Lactuca sativa* | *LsIAA16* | Lsat_1_v5_gn_9_10001 |
| *Lactuca sativa* | *LsARF5a* | Lsat_1_v5_gn_2_12381 |
| *Lactuca sativa* | *LsARF5b* | Lsat_1_v5_gn_9_46321 |
| *Lactuca sativa* | *LsGH3.1a* | Lsat_1_v5_gn_5_116820 |
| *Lactuca sativa* | *LsGH3.1b* | Lsat_1_v5_gn_2_84761 |
| *Lactuca sativa* | *LsGH3.1c* | Lsat_1_v5_gn_4_162960 |
| *Lactuca sativa* | *LsSAUR12* | Lsat_1_v5_gn_8_126641 |
| *Lactuca sativa* | *LsSAUR45* | Lsat_1_v5_gn_9_20900 |
| *Lactuca sativa* | *LsSAUR70* | Lsat_1_v5_gn_8_71421 |
| *Lactuca sativa* | *LsSAUR72* | Lsat_1_v5_gn_7_70721 |
| *Lactuca sativa* | *LsSAUR22* | Lsat_1_v5_gn_3_45581 |
| *Lactuca sativa* | *LsSAUR59* | Lsat_1_v5_gn_3_2200 |
| *Lactuca sativa* | *LsREV1* | Lsat_1_v5_gn_2_100140 |
| *Lactuca sativa* | *LsREV2* | Lsat_1_v5_gn_6_45641 |
| *Lactuca sativa* | *LsAS1* | Lsat_1_v5_gn_4_153581 |
| *Lactuca sativa* | *LsKAN1* | Lsat_1_v5_gn_3_67301 |
| *Lactuca sativa* | *LsKAN2* | Lsat_1_v5_gn_3_18021 |
| *Lactuca sativa* | *LsYAB1a* | Lsat_1_v5_gn_7_9041 |
| *Lactuca sativa* | *LsYAB1b* | Lsat_1_v5_gn_3_128981 |
| *Lactuca sativa* | *LsYAB1c* | Lsat_1_v5_gn_3_3040 |
| *Lactuca sativa* | *LsYAB3* | Lsat_1_v5_gn_6_2400 |
| *Arabidopsis thaliana* | *AtTPL* | AT1G15750 |
| *Arabidopsis thaliana* | *AtTPR1* | AT1G80490 |
| *Arabidopsis thaliana* | *AtTPR2* | AT3G16830 |
| *Arabidopsis thaliana* | *AtTPR3* | AT5G27030 |
| *Arabidopsis thaliana* | *AtTPR4* | AT3G15880 |
| *Arabidopsis thaliana* | *AtTCP2* | AT4G18390 |
| *Arabidopsis thaliana* | *AtTCP3* | AT1G53230 |
| *Arabidopsis thaliana* | *AtTCP4* | AT3G15030 |
| *Arabidopsis thaliana* | *AtTCP5* | AT5G60970 |
| *Arabidopsis thaliana* | *AtTCP10* | AT2G31070 |
| *Arabidopsis thaliana* | *AtTCP13* | AT3G02150 |
| *Arabidopsis thaliana* | *AtTCP17* | AT5G08070 |
| *Arabidopsis thaliana* | *AtTCP24* | AT1G30210 |
| *Antirrhinum majus* | *AmCIN* | Am03g11310 |
| *Antirrhinum majus* | *AmCIN-like* | Am01g49070 |
| *Antirrhinum majus* | *AmCIN-like* | Am02g02040 |
| *Antirrhinum majus* | *AmCIN-like* | Am02g04490 |
| *Antirrhinum majus* | *AmCIN-like* | Am04g25320 |
| *Antirrhinum majus* | *AmCIN-like* | Am06g33680 |
| *Antirrhinum majus* | *AmCIN-like* | Am07g11420 |
| *Antirrhinum majus* | *AmCIN-like* | Am08g21950 |
